# Integrated design for rural microgrids in Egypt incorporating hydrogen energy and vehicle-to-grid technology

**DOI:** 10.1038/s41598-026-67972-2

**Published:** 2026-09-09

**Authors:** M. Shamy Sayed, M. H. Alham, Doaa Khalil Ibrahim

**Affiliations:** Electrical Power Engineering Department, Faculty of Engineering, Cairo University, Giza, Cairo 12613 Egypt

**Keywords:** Electric vehicle, Hydrogen energy storage systems, Hydrogen vehicle, Loss-of-power-supply probability, Renewable energy sources, Vehicle-to-grid, Energy science and technology, Engineering

## Abstract

Green hydrogen (H_2_) has gained considerable attention as a cornerstone of a zero-emission future. The global transportation sector is shifting from gasoline-powered vehicles toward electric vehicles (EVs) and hydrogen vehicles (HVs) to reduce emissions. This paper investigates a microgrid (MG) model comprising photovoltaic panels, wind turbines, batteries, and hydrogen energy storage systems integrated with vehicle-to-grid (V2G) systems for EVs and HVs. The stochastic behavior of both EVs and HVs is characterized using probability density functions to capture vehicle uncertainty. A double-layer optimization model is proposed to enhance the reliability, cost-effectiveness, and sustainability of MGs. The upper layer employs the non-dominated sorting genetic algorithm II to optimize V2G scheduling, thereby minimizing MG load variance and total operational costs for EV and HV owners. The lower-layer approach is a multi-objective optimization problem that minimizes system costs over the entire life cycle while maximizing MG reliability, solved using the Gurobi optimizer. The framework is validated for an islanded MG in El-Kharga Oasis, Egypt, as an off-grid community with high renewable energy potential. Various scenarios are examined for EV and HV behavior, with the optimal cost achieved when G2V and V2G modes are applied to both HVs and EVs, resulting in a 12% cost reduction relative to the scenario without V2G. Further analysis is conducted to examine the impact of the loss-of-power-supply probability (LPSP) value on system economics. Results indicate that implementing an LPSP constraint of 0.05 reduces the annualized total cost by around 21.2% compared to the baseline case without the LPSP constraint.

## Introduction

Microgrids (MGs) are increasingly recognized as a viable framework for integrating renewable energy sources (RES), such as photovoltaic (PV) systems and wind turbines (WT), and incorporating energy storage systems (ESS). Nowadays, the integration of PV systems extends beyond stationary MG applications. Recent studies have examined PV deployment strategies using optimization-based frameworks for hybrid configurations that yield larger discounted life-cycle net benefits, underscoring the growing role of PV technology across both stationary and mobile energy systems^1^. Recently, the hydrogen energy storage system (H_2_ESS), which includes an electrolyzer (EL), fuel cell (FC), and hydrogen tank (HT), has received significant attention due to its cleanliness, efficiency, and large storage capacity^2,3^. It will be easier to incorporate hydrogen (H_2_) generation and use into an energy storage system as more people start using H_2_ technology and the number of installed WT and PV systems grows. H_2_ technology could make MG operations more robust and cost-effective while reducing the power curtailment rates of wind and solar systems^4^.

Currently, substantial research is directed toward the techno-economic analysis of renewable-based isolated MGs with diverse resources while incorporating H_2_ as an energy storage medium. For example, the investigation of an MG for a university campus has been addressed using HOMER Pro optimization in^5^. It compared three configurations that include PV systems, WTs, batteries, and green H_2_ and concluded that the hybrid system combining PV, WTs, batteries, and green H_2_ is the most economically viable. Another study is introduced in^6^ and evaluated across three geographically and economically distinct zones in Bangladesh: Anwara, Matarbari, and Pabna. The proposed framework applies the mixing and exploring algorithm (MEXA)—a recently developed meta-heuristic optimization algorithm—to determine the optimal sizing and operational strategy of a hybrid MG system comprising PV panels, WTs, battery storage, EL, and a diesel generator (DG). A comprehensive comparison of isolated solar hybrid systems that considered all three major backup options: H_2_, battery, and DG is presented in^7^. The enhanced particle swarm optimization (PSO) algorithm is applied for techno-economic-environmental analysis and the optimal design of solar/H_2_, solar/battery, and solar/diesel systems. Although the solar/diesel system may appear economically attractive at first, its considerable emissions and sensitivity to fuel price fluctuations diminish its long-term sustainability. Conversely, solar/battery systems deliver superior environmental performance, whereas solar/H_2_ systems provide greater long-term reliability despite the elevated capital costs. For the co-production of electricity and green H_2_, a techno-economic analysis of an integrated system combining offshore wind turbines (OWT) and floating solar photovoltaic (FPV) technology is also conducted in^8^ by utilizing dung beetle optimization (DBO). It has been deduced that the hybrid FPV/OWT system is more cost-effective.

Moreover, an optimal energy management strategy is introduced in^9^ for a grid-connected MG. It significantly reduced the cost of energy for the H_2_ production system from $0.435/kWh to $0.207/kWh by prioritizing PV usage and battery storage to supply the electrolyzer before drawing from the grid. The hybrid renewable MGs integrating H_2_ and battery storage for residential buildings in Canada have been developed in^10^ under both on-grid and off-grid conditions. It demonstrated that H_2_–battery co-storage enhances system reliability and utilization of renewable energy. Beyond reliability, managing ESS profoundly impacts MG operations, risk management, and market architectures. Operationally,^11^ demonstrated that coordinated engine-storage control is vital to smooth electromechanical transient mismatches in isolated MGs facing severe pulse loads. Applying a multi-objective framework with conditional value-at-risk in^12^ ensures that H_2_ storage mitigates the financial and carbon risks of renewable volatility. Concerning the shift from localized assets to shared infrastructure, the Stackelberg-game-based cloud storage framework proposed in^13^ proves that multi-energy capacity leasing reduces individual capital costs while maximizing global resource efficiency.

None of the aforementioned studies^5–13^ considered incorporating electric vehicles (EVs) or hydrogen fuel cell vehicles (HVs) into their proposed frameworks.

Many countries are adopting EV technology and establishing charging infrastructure to mitigate pollution. Along with EVs, HVs have also gained commercial popularity globally^14^. Hydrogen refueling for HVs can be completed within 3–5 min, significantly faster than EV charging, with implications for station design and energy system integration^15^. Over the past decade, researchers have conducted extensive studies on managing the operation of MGs integrated with EVs and HVs. The feasibility of green H_2_-based MGs for powering fuel cell buses in Fiji is evaluated in^16^, comparing grid-connected/off-grid configurations using solar-wind hybrids. A novel energy management system and Kepler optimization algorithm (KOA) are applied in^17^ to optimally manage the integrated MG with plug-in hybrid EVs across three charging modes (uncoordinated, coordinated, and smart charging) to minimize both operational costs and environmental emissions under uncertainty. The MG investigated in^17^ is connected to the grid and includes WT, PV, micro turbine (MT), FC, and storage batteries. An optimal sizing for isolated MGs is developed in^18^ by accounting for the stochastic behavior of EV users and uncertainty in renewable energy sources and home load demand. It solved the capacity configuration problem using a stochastic programming approach to determine the optimal component sizes (PV, WT, battery, EV fleet) to minimize the total system cost and improve MG resilience. Beyond individual MG scheduling, the aggregation of EVs into virtual entities participating in electricity markets has gained considerable attention. A bidding method for EV aggregators (EVAs) in the flexible ramping product trading market is proposed in^19^, where a bi-level optimization model is developed to coordinate incentive pricing and charging strategies for both plug-in EVs and battery-swapping EVs, simultaneously addressing the conflicting objectives of EVAs and EV owners while enhancing overall grid flexibility. A strategy was proposed in^20^ for scheduling the charging/discharging of EVs in MGs, incorporating user satisfaction alongside load variance reduction using chaotic perturbation and multi-role particle strategies.

Furthermore, a Vehicle-to-Grid (V2G) approach is explored in the MG context in^21^, demonstrating how coordinated EV discharging can support grid stability and reduce overall operating costs. An optimal design and three-level stochastic energy management of an interconnected MG with an HV refueling station are introduced in^22^. This study proposes a cost-minimization framework that accounts for the uncertainty of renewable generation, load demand, and hydrogen consumption, offering an effective strategy for integrating H_2_ infrastructure into modern MGs. A stochastic optimal scheduling model has been proposed in^23^ for a renewable-based MG with FCs and EV–V2G integration using the modified fluid search optimization (MFSO) algorithm. It succeeded in enhancing flexibility and achieving lower operating costs. A hierarchical co-optimal planning framework to manage MGs by integrating H_2_ESS and V2G from EVs, alongside HVs, is developed in^24^. Using the non-dominated sorting genetic algorithm II (NSGA-II), the framework successfully minimized the total costs for MGs (investment and operation) while leveraging the synergistic effect of H_2_ESS, V2G (EV), and HV charging to handle renewable energy and load uncertainties. H_2_-based hybrid storage and EV batteries employing V2G technology are investigated in the stochastic optimum design of a rural MG in^25^. The analysis considered the stochastic operation of EVs and HVs to reduce total life cycle cost and maximize system reliability.

To manage competing operational objectives and system constraints, multi-layer and bi-level optimization architectures have been actively explored in recent literature. For instance, a decentralized two-layer optimization framework is proposed in^26^ to enhance MG resilience. In such a setup, the first layer independently schedules individual MGs to ensure data privacy. In contrast, the second layer coordinates the distribution network through feeder reconfiguration and the strategic dispatch of network nodes. This configuration relies on a robust optimization approach to actively counter the uncertainties governing load demand, renewable generation, and electric bus mobility constraints. Similarly, a bi-level optimization model for grid-connected MGs featuring H_2_ESS is developed in^27^. In this model, the upper level minimizes global MG operation and grid-trading costs, and the lower level minimizes localized operational and maintenance costs of H2ESS components for RES variations. Moving towards market integration^28^ introduced a risk-averse bi-level day-ahead planning formulation for a green H_2_ system integrated with an H_2_ fueling station to service HVs. In such a framework, the upper level maximizes the total system profit through energy market trading and resource allocation. On the other hand, its lower level minimizes consumer costs by price-based responsive loads under the risk-averse management of renewable energy output and market price uncertainties. The general algebraic modeling system (GAMS), as a high-level modeling system, is utilized in^26^-^28^.

Table 1 summarizes some published studies regarding components, incorporating EV/HV, considering uncertainties, and the applied solutions. For comparison, the final row outlines the features and contributions of the present study across the same items. As evidenced by the comparative analysis in Table 1, only two studies^24,25^ incorporated both EVs and HVs; however, neither provided comprehensive coverage of the uncertainties governing vehicle behavior. Few studies applied the V2G mode for both EVs and HVs. To address this research gap, this work models hybrid renewable energy systems (HRESs) integrated with H_2_ and battery storage alongside V2G capabilities for EVs and HVs. The study analyzes their effects on enhancing the reliability, cost-effectiveness, and sustainability of MGs.

**Table 1:** Summary of some previous studies and the contribution of this study.

References	System components	EV	HV	V2G	EV uncertainty	HV uncertainty	Solution methods	Multi-layer	Multi-objective
^5^	PV, WT, Battery, HT, EL, FC	–	–	–	–	–	HOMER Pro	–	–
^6^	PV, WT, Battery, DG, EL	–	–	–	–	–	MEXA	–	✓
^7^	PV, Battery, DG, EL, HT, FC	–	–	–	–	–	Enhanced PSO	–	✓
^8^	PV, WT, EL, FC, HT	–	–	–	–	–	DBO	–	✓
^9^	PV, WT, FC, EL, HT, Battery, Grid	–	–	–	–	–	Gurobi	–	–
^10^	PV, WT, Battery, HT, EL, FC, DG	–	–	–	–	–	HOMER Pro	–	–
^16^	PV, WT, Battery, HT, EL, Grid	–	✓	–	–	–	HOMER Pro	–	–
^17^	PV, WT, Battery, MT, FC, Grid	✓	–	–	–	–	KOA	–	✓
^18^	PV, WT, Battery	✓	–	✓	–	–	Gurobi	–	✓
^22^	PV, WT, FC, EL, HT, Grid	–	✓	✓	-	✓	PSO	Three levels of energy management	
^23^	PV, WT, FC, MT	✓	–	✓	✓	-	MFSO	–	–
^24^	PV, WT, FC, EL, HT, Grid	✓	✓	✓	✓	–	NSGA-II & Gurobi	✓	✓
^25^	PV, WT, FC, EL, HT, Battery	✓	✓	✓	–	–	Gurobi	–	✓
^26^	PV, WT, FC, EL, HT, Battery, Gas turbine	✓	–	–	✓	–	GAMS	✓	–
^27^	PV, WT, DG, EL, HT, FC, Grid	–	–	–	–	–	GAMS	✓	–
^28^	PV, WT, FC, EL, HT, Battery	–	✓	–	–	–	GAMS	✓	–
This article	PV, WT, FC, EL, HT, Battery	✓	✓	✓	✓	✓	NSGA-II & Gurobi	✓	✓

A cornerstone of Egypt's Integrated Sustainable Energy Strategy 2035 is an ambitious commitment to derive 42% of the national electricity generation from renewable sources, namely wind, solar, and hydropower. Lately, Egypt has announced plans to produce green H_2_ and become a major exporter, aiming to capture 5% of the global hydrogen market by 2030 and 8% by 2040. It is made possible by the launch of Egypt's green hydrogen strategy at COP27^29^. Consequently, this extensive national focus on green H_2_ production is expected to drive the long-term expansion of private HVs. Nowadays, the private EV market in Egypt is witnessing rapid acceleration. Therefore, Egypt has become the leading EV market in Africa with total licensed EVs exceeding 7,200 units by mid-2024 and recording 244.3% market growth in Q1 2026^30^. Therefore, this study targets the optimal planning, sizing, and operation of rural MGs in Egypt, integrating HVs and EVs.

The proposed architecture is distinguished from existing literature by the simultaneous integration of two distinct mobile energy assets, EVs and HVs, within a single islanded rural MG under a unified bidirectional G2V/V2G operation framework. Unlike prior studies that address EV or HV in isolation, this work develops coupled stochastic models for both asset types, each with independent uncertainty characterization using probability density functions (PDFs). It integrates them within a double-layer multi-objective optimization structure that explicitly links V2G/G2V scheduling decisions to long-term MG sizing outcomes. Accordingly, the main goals of the study can be summarized as follows:

- Modeling different flexible resources, like H_2_ energy and V2G technology for both EVs and HVs, to make the MG system more reliable, cost-effective, and emission-free.
- The uncertainties of arrival time at the refueling station, driving distance, and initial state of health of HVs are considered and represented using appropriate types of PDFs.
- Furthermore, the uncertainties of arrival and departure times of EVs to homes and driving distance are considered using PDFs.
- The influences of V2G mode for EVs and HVs, considering degradation and different Loss-of-Power-Supply Probability (LPSP) values on optimal sizing, are extensively examined.
- A double-layer optimization model is performed, where both upper and lower layers are multi-objective functions. The upper-layer model includes V2G optimal scheduling that tries to minimize both the MG load variance and the total costs incurred by EV and HV owners. It aims to make profits for investors in MGs, EVs, and HVs. The lower-layer model aims to lower both the Annualized Total Cost (ATC) and LPSP. The upper layer is solved using NSGA-II, and the lower layer is solved using the Gurobi solver version 12.0.1 integrated into MATLAB 2022b.

It is worth noting that the solver selection above is not a standalone contribution. Still, a deliberate choice should be matched to each layer's mathematical structure. The upper layer is non-convex and stochastic, precluding the use of exact solvers. In contrast, the lower layer is a Mixed-Integer Quadratically Constrained Programming (MIQCP) problem, for which an exact solver guarantees the proven global optimum. This pairing is what ensures the reliability of the framework's results.

The remainder of this paper is organized as follows: "Case study description" section describes the study location and its natural resources. "System modelling" section presents the mathematical modeling of the MG components. "Problem formulation" section formulates the proposed objective functions and the associated constraints. Then, "Results and discussion" section presents and discusses the obtained results. Finally, the summary of the main findings is listed in "Conclusions" section.

## Case study description

**Fig. 1:**
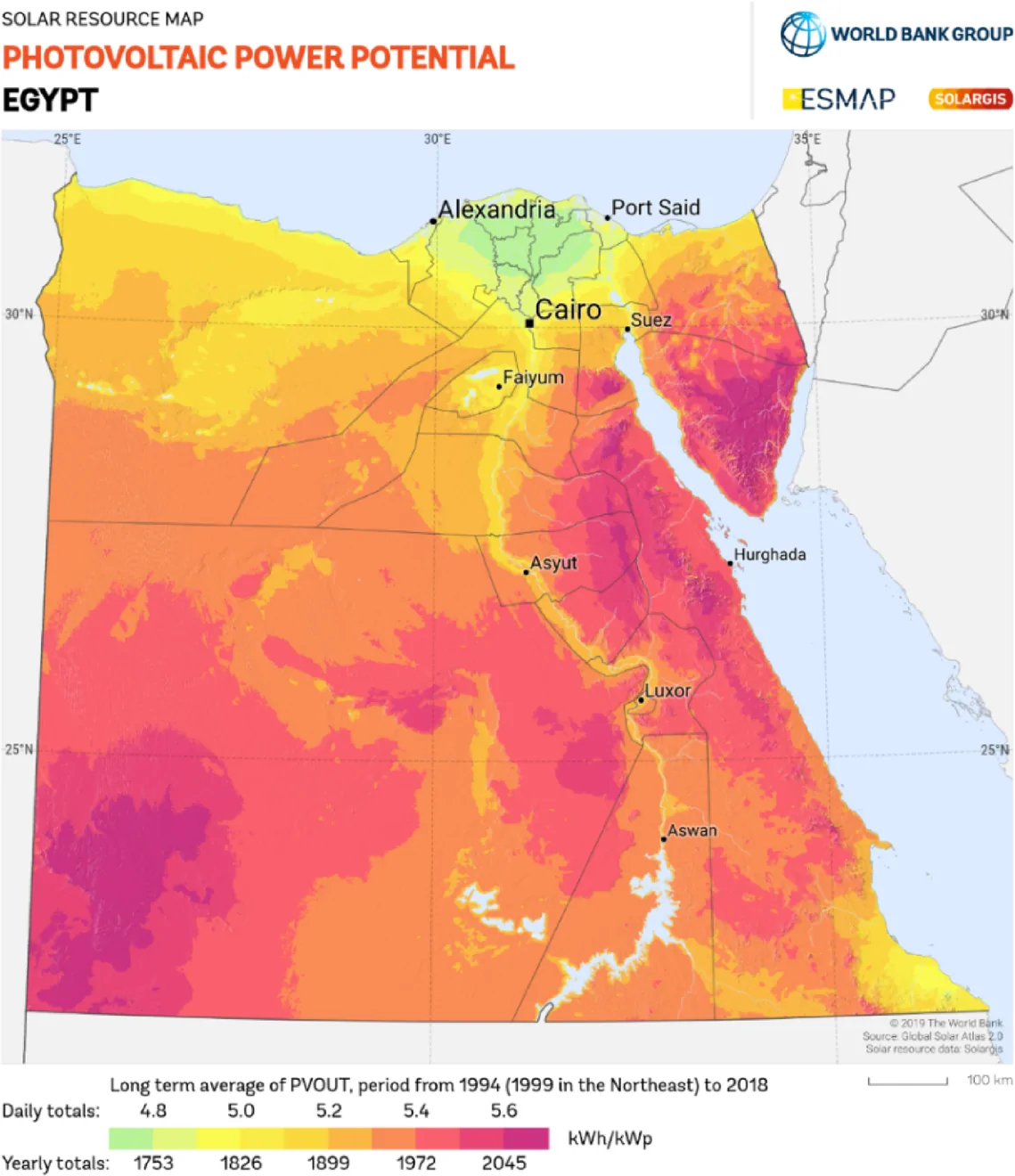
Photovoltaic power potential map of Egypt^31^. The map is obtained from the Global Solar Atlas 2.0, a free, web-based application developed and operated by Solargis s.r.o. on behalf of the World Bank Group, utilizing Solargis data, with funding provided by the Energy Sector Management Assistance Program (ESMAP). For additional information: https://globalsolaratlas.info.

**Fig. 2:**
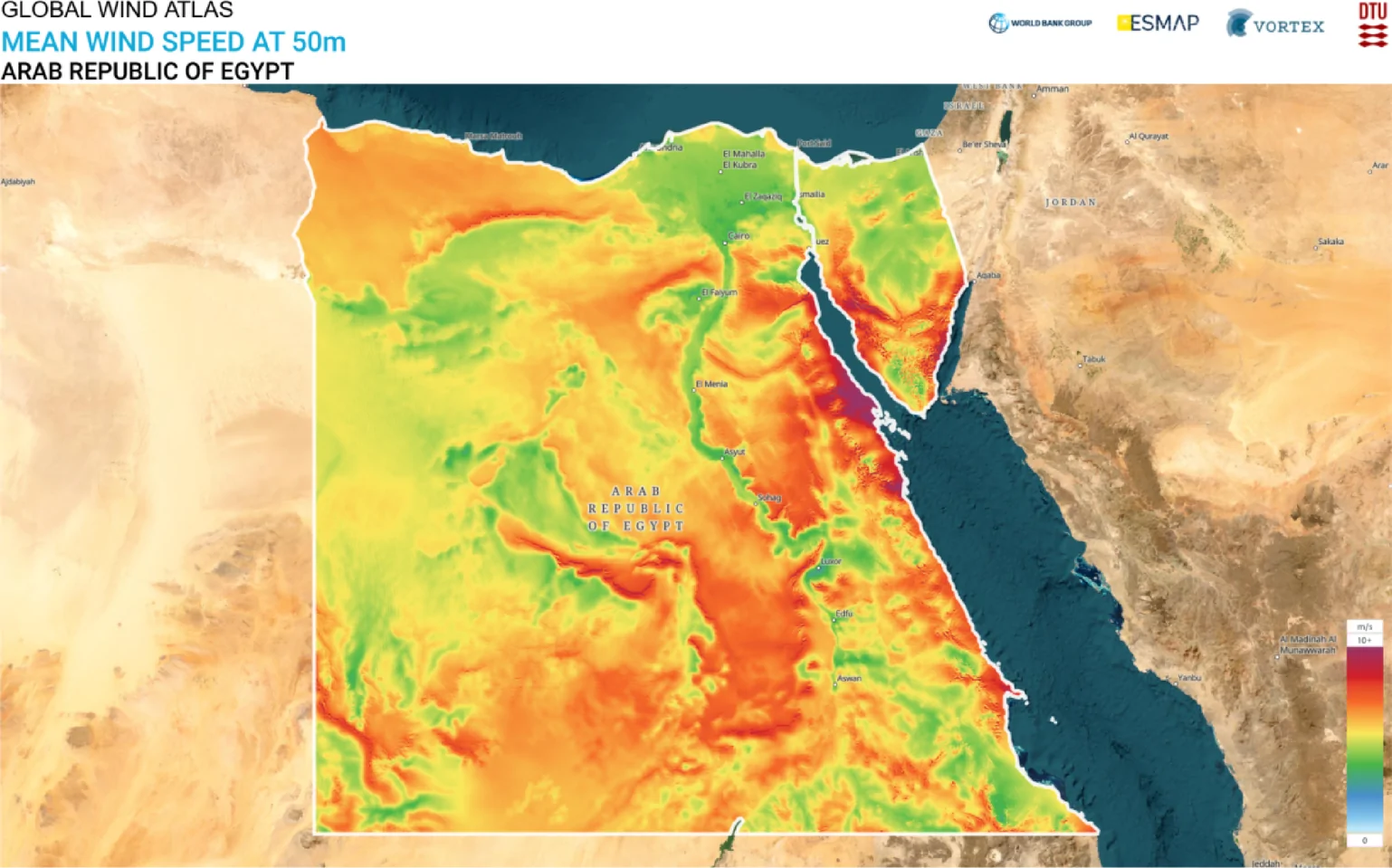
Mean wind speed map of Egypt at 50 m hub height^32^. The map is obtained from the Global Wind Atlas version 4.0, a free, web-based application developed, owned, and operated by the Technical University of Denmark (DTU). The Global Wind Atlas version 4.0 is released in partnership with the World Bank Group, utilizing data provided by Vortex, using funding provided by the Energy Sector Management Assistance Program (ESMAP). For additional information: https://globalwindatlas.info.

Egypt exhibits substantial solar energy and wind potential across large parts of its territory, as illustrated by its photovoltaic power potential map in Fig. 1^31^ and its mean wind speed map at 50 m hub height in Fig. 2^32^. This study evaluates the feasibility of an off-grid MG integrating HRESs (PV and WT), H2ESS, and battery storage to supply residential buildings in rural areas in Egypt. El-Kharga Oasis, Egypt, is selected as a case study. El-Kharga Oasis, situated in the remote southwest of the Nile Valley (25.441188° N, 30.521838° E), is an ideal candidate for off-grid renewable energy deployment.

### Natural resources

**Fig. 3:**
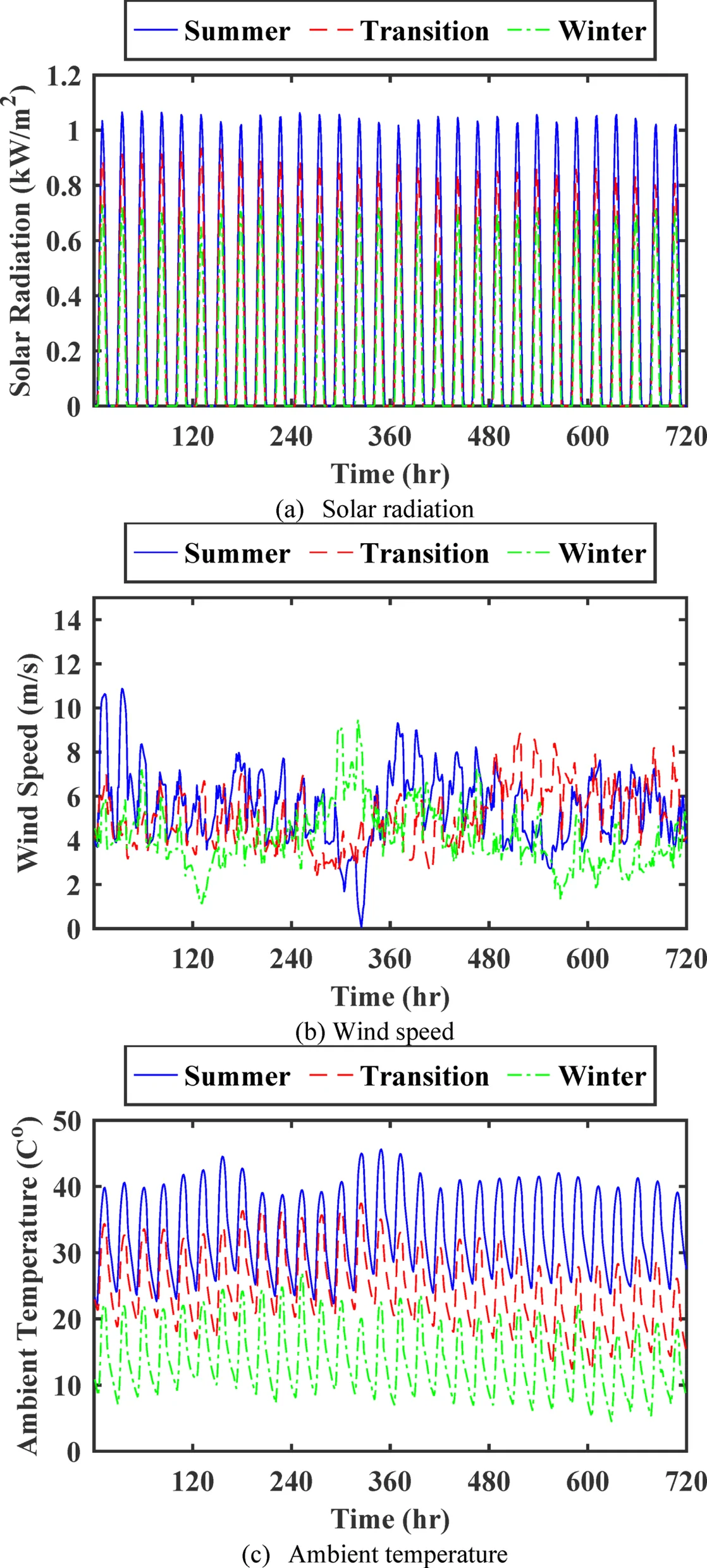
Natural resources for El-Kharga Oasis, Egypt, as the selected location.

El-Kharga Oasis is the studied region that falls within areas of moderate wind speed and favorable solar irradiance, supporting the viability of the proposed hybrid system. To enhance practical applicability, analyses were conducted using PV output, WT output, and residential electric load demand data for three representative months that collectively capture the key seasonal variations of the study region: the transition season, summer, and winter. Figure 3 presents the corresponding solar radiation, wind speed at 10 m hub height, and ambient temperature profiles for a representative 30-day period (720 h) for each season, sourced from^33^.

### Residential load

**Fig. 4:**
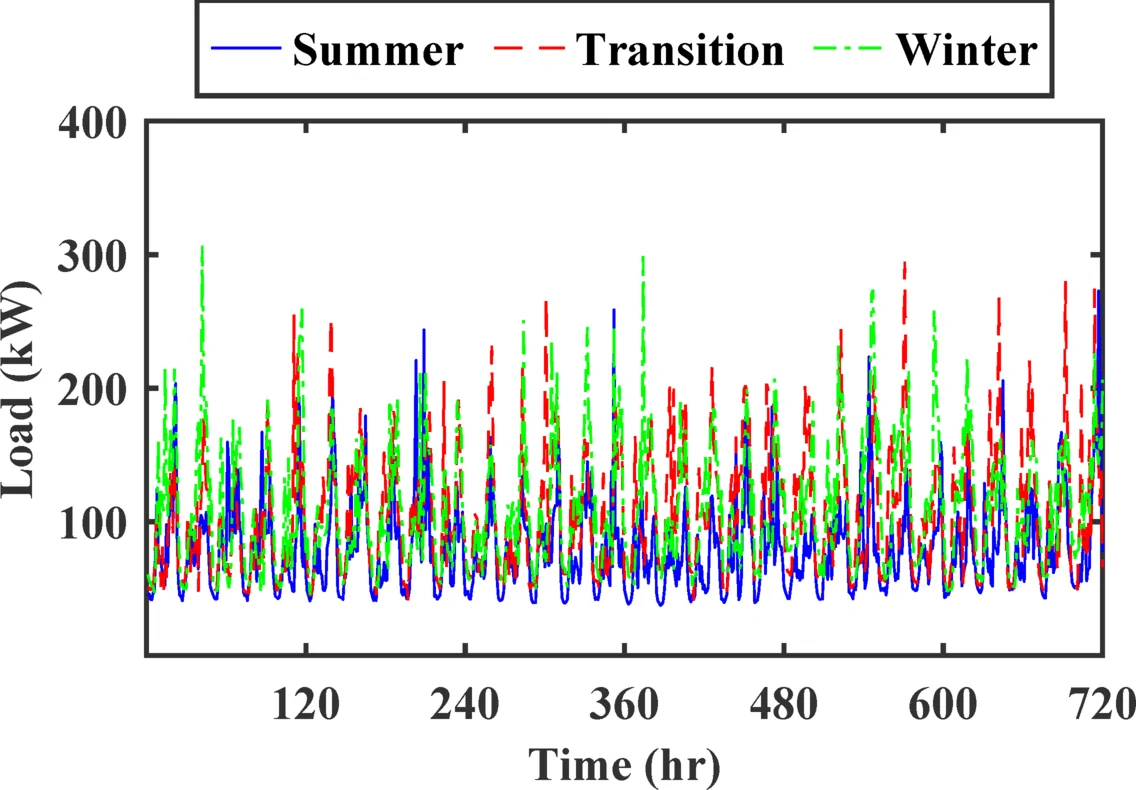
Electric load on a typical month in different seasons.

The hourly electric load demand profiles for the transition, summer, and winter seasons are presented in Fig. 4, covering 240 apartments distributed across 12 residential buildings. The dataset captures six distinct residential consumption behaviors over a range from low to high demand levels, as derived from^34^. Out of the 240 apartments, 30 are reserved for managers. These managers work at nearby factories and outside the MG. Because they are located nearby, the residents have short and highly predictable daily commutes. To match realistic vehicle ownership trends, the MG model includes 20 EVs and 10 HVs assigned to managers. The system uses home charging for private EVs. This setup avoids putting strain on the public grid. Meanwhile, a small public hydrogen refueling station is placed inside the compound to service the HVs.

## System modelling

**Fig. 5:**
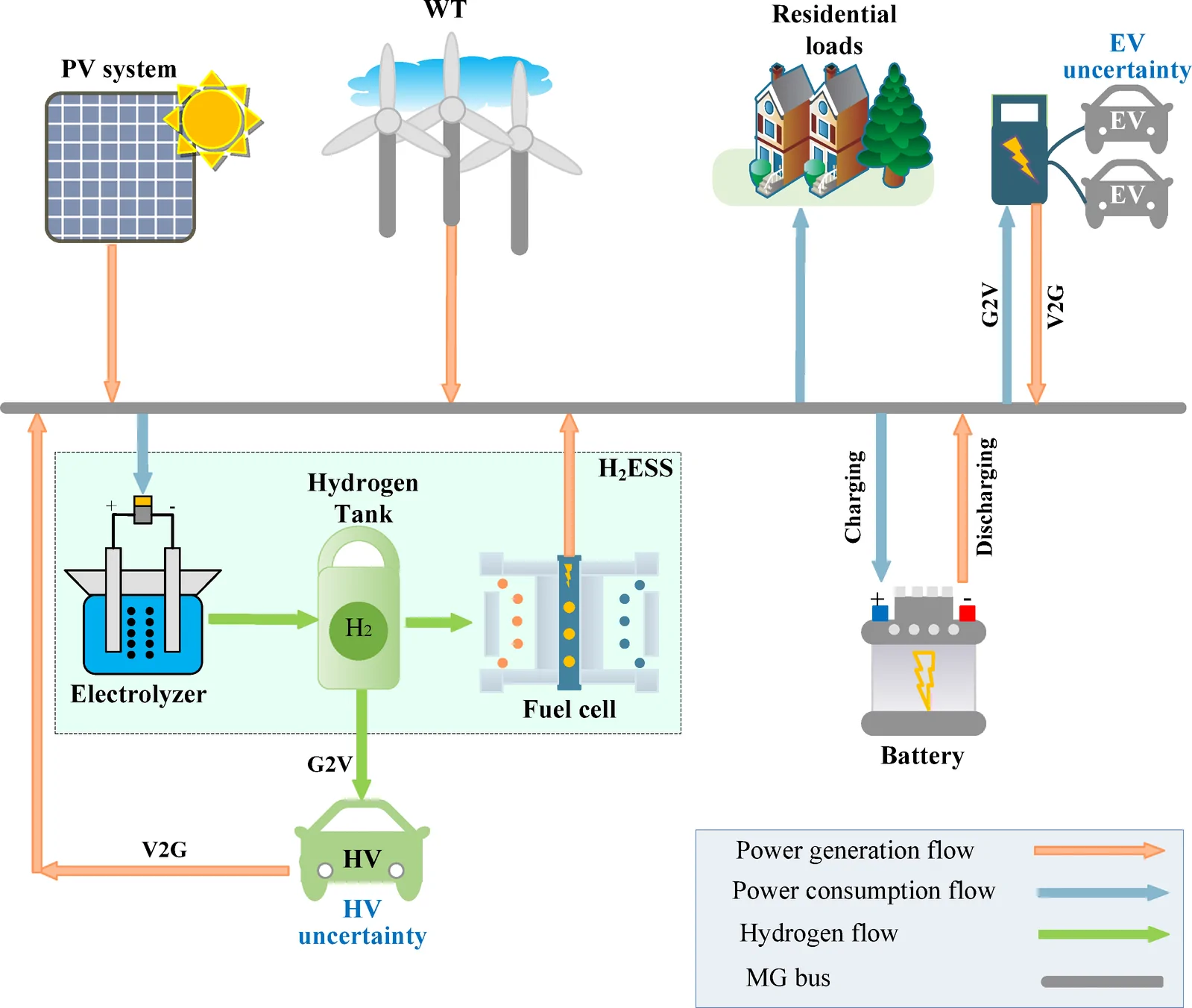
System architecture of the investigated MG under study.

This research indicates the optimal sizing of a rural MG system. The system comprises a WT, PV panels, a battery (BT), hydrogen-based storage, EVs, and HVs. Figure 5 illustrates the isolated MG system, showing the interconnections among components and the direction of energy flow for each element. The study omitted detailed modeling of power converters, as their high conversion efficiency makes the associated losses negligible. Hydrogen produced from RES is supplied to HVs and FC. EVs and HVs operate in two scheduling modes: G2V and V2G. In G2V mode, EVs and HVs function as electrical loads, drawing power from the grid. The mathematical modeling for subsystems is described in the following sections.

### PV model

The actual power production of PV panels (${\mathrm{P}}_{\mathrm{t}}^{\mathrm{P}\mathrm{V}-\mathrm{o}\mathrm{u}\mathrm{t}}$) at time *t* is determined by the conditions of the solar irradiation level (${G}_{t}$) and ambient temperature (${T}_{amb, t}$), as presented in Eq. (1)^6^.

1 $$ P_{t}^{PV - out} = N^{PV} \cdot P_{PV,r} \cdot \eta^{PV} \cdot \frac{{G_{t} }}{{G_{STC} }} \cdot \left[ {1 + \varphi \cdot \left( {T_{amb, t} - T_{STC} } \right)} \right] $$

where ${P}_{PV,r}$ is the rated power of a single PV panel under standard test conditions (STC). Solar irradiance intensity and panel temperature under STC are denoted by ${G}_{STC}$ and ${T}_{STC}$, respectively. ${N}^{PV}$ is the number of PV panels; ${\eta }^{PV}$ is the power loss coefficient that takes into account factors such as shading, dirt, and temperature effects.

The technical characteristics and PV parameters are tabulated in Table 2, including the temperature coefficient (*φ*).

**Table 2:** System parameters^22,24,25,40^.

Parameters of generation systems						
PV		$${P}_{PV,r}$$	$${\eta }^{PV}$$	$${G}_{STC}$$	φ	$${T}_{STC}$$
		1 kW	0.8	1 kW/m^2^	− 0.005$${{^\circ{\rm C} }}^{-1}$$	25$${^\circ{\rm C} }$$
		$${C}_{PV}^{CI}$$	$${C}_{PV}^{OM}$$	$${A}^{PV}$$	Life span	
		492 $/kW	4.92 $/kW	1.95 m^2^	20 years	
WT		$${P}_{WT,r}$$	$${V}_{ci}$$,$${V}_{co}$$	$${V}_{r}$$	$${H}_{WT}$$	$${H}_{ref}$$
		5 kW	2.5 m/s, 25 m/s	12 m/s	40 m	10 m
		$${C}_{WT}^{CI}$$	$${C}_{WT}^{OM}$$	D	Life span	
		563 $/kW	11.25 $/kW	5.15 m	20 years	
Parameters of vehicles						
EV		$${N}^{EV}$$	$${P}^{ch}$$,$${P}^{dis}$$	$${\mu}_{d}^{EV}$$,$${\delta}_{d}$$	$${\mu}_{ta}^{EV}$$,$${\delta}_{{t}_{a}}$$	$${\mu}_{td}^{EV}$$,$${\delta}_{{t}_{d}}$$
		20 EVs	5 kW, -5 kW	3.2 km, 0.88 km	18.8 h, 3.5 h	7.97 h, 0.66 h
		$${Q}^{EV}$$,$${\Delta E}^{EV}$$	*n*	$${\eta }^{EV}$$	$${c}^{Bat}$$	$$SO{C}^{max}$$,$$SO{C}^{min}$$
		30 kWh, 0.15 kWh/km	2151 cycles	90%	17,780 $	80%, 20%
HV		$${N}^{HV}$$	$${m}^{HV}$$	$${\eta }^{HV}$$	$${\mu}_{{T}_{a}}$$	$${\delta}_{{T}_{a}}$$
		10 HVs	5.65 kg	60%	8.3 h	3 h
		$${\mu}_{{m}_{in}}$$	$${\delta}_{{m}_{in}}$$	$${d}_{max}^{HV}$$	$${c}^{FC}$$	$${\Delta E}^{HV}$$
		35%	25%	160,934 km	14,100 $	0.283 kWh/km
Parameters of storage systems						
BT		$${Q}_{BT}$$	$${SOC}_{max-BT}$$	$${SOC}_{min-BT}$$	$${P}_{BT,max}^{Dch}$$	$${P}_{BT,max}^{Ch}$$
		4 kWh	80%	20%	6.91 kW	4.61 kW
		$${\eta }^{BT,Dch}$$	$${\eta }^{BT,Ch}$$	$${C}_{BT}^{CI}$$	$${C}_{BT}^{OM}$$	Lifetime
		90%	80%	300 $/unit	10 $/unit	5 years
H_2_ESS	EL	$${P}_{max}^{EL}$$	$${\eta }^{EL}$$	$${C}_{EL}^{CI}$$	$${C}_{EL}^{OM}$$	Life span
		6.2 kW	55%	311 $/kg	12.45 $/kW/year	10 years
	FC	$${P}_{max}^{FC}$$	$${\eta }^{FC}$$	$${C}_{FC}^{CI}$$	$${C}_{FC}^{OM}$$	Life span
		6 kW	45%	450 $/kg	18 $/kW/year	5 years
	HT	$${SOH}_{max}$$,$${SOH}_{min}$$	$${\eta }^{HT}$$,$${m}_{r}^{HT}$$	HHV	$${C}_{HT}^{CI}$$,$${C}_{HT}^{OM}$$	Life span
		90%, 10%	95%, 1 kg	39.7 kWh/kg	423 $/kg, 4.23 $/kg/year	20 years

### Wind turbine model

The output power of a WT is determined by WT characteristics and the wind speed at hub height (${H}_{WT}$), which is estimated as follows^6^:

2 $$ V_{WT,t} = V_{ref,t} \cdot \left( {\frac{{H_{WT} }}{{H_{ref} }}} \right)^{\alpha } $$

where ${\mathrm{V}}_{\mathrm{W}\mathrm{T},\mathrm{t}}$ and ${\mathrm{V}}_{\mathrm{r}\mathrm{e}\mathrm{f},\mathrm{t}}$ are the wind speeds measured at the WT hub height and at the reference height (${\mathrm{H}}_{\mathrm{r}\mathrm{e}\mathrm{f}}$) at time *t*, respectively. The friction coefficient (*α *)*,* which is affected by ground roughness, is assumed to be 1/7 in this study.

For ${N}^{WT}$ as the number of WTs, the output power (${\mathrm{P}}_{\mathrm{t}}^{\mathrm{W}\mathrm{T}, \mathrm{o}\mathrm{u}\mathrm{t}}$) depends on the real-time wind speed (${\mathrm{V}}_{\mathrm{W}\mathrm{T},\mathrm{t}}$) at hub height as calculated in Eq. (3), where ${P}_{WT,r}$ refers to the rated wind power; ${V}_{ci}$, ${V}_{co}$, and ${V}_{r}$ represent the cut-in wind speed, cut-out wind speed, and rated wind speed, respectively^35^.

3 $${P}_{t}^{WT,out}={N}^{WT}\times \left\{\begin{array}{l}0 { V}_{WT,t}<{V}_{ci} or {V}_{WT,t}>{V}_{co} \\ {P}_{WT,r}\times \frac{{{(V}_{WT,t})}^{3}-{{ (V}_{ci})}^{3}}{{{(V}_{r})}^{3}- {{ (V}_{ci})}^{3}} {V}_{ci}\le {V}_{WT,t}\le {V}_{r}\\ {P}_{WT,r} {V}_{r}\le {V}_{WT,t}\le {V}_{co}\end{array}\right.$$

### Battery model

The safe operation of the battery is ensured by its operational limits. Overcharging and discharging can also harm the battery, so Eq. (4) is considered for the maximum and minimum SOC limits. The charging power (${P}_{BT,t}^{Ch}$) and discharging power (${P}_{BT,t}^{Dch}$) must remain within the maximum and minimum constraints specified in Eq. (5) for the entire simulation time (*T*).

4 $$ SOC_{\min - BT} \le SOC_{t} \le SOC_{\max - BT} \forall t \in T $$

5 $$ \left\{ {\begin{array}{*{20}c} {P_{BT,\min }^{Dch} \le P_{BT,t}^{Dch} \le P_{BT,\max }^{Dch} } \\ {P_{BT,\min }^{Ch} \le P_{BT,t}^{Ch} \le P_{BT,\max }^{Ch} } \\ \end{array} } \right. $$

Considering the power lost during discharging and charging (${\delta}_{BT}^{Dsp}$ equals 0.006%), Eq. (6) calculates the SOC balance at time *t*, where ${\eta }^{BT,Ch}$, and ${\eta }^{BT,Dch}$ are the battery efficiencies for charging and discharging, respectively, and ${Q}_{BT}$ is the rated capacity of the battery. To prevent simultaneously charging and discharging the battery, Eq. (7) is imposed^9^. The battery parameters are presented in Table 2.

6 $$ SOC_{t} = SOC_{t - 1} + \frac{{\eta^{BT,Ch} \times P_{BT,t}^{Ch} \times \Delta t}}{{Q_{BT} }} - \frac{{P_{BT,t}^{Dch} \times \Delta t}}{{\eta^{BT,Dch} \times Q_{BT} }} - \delta_{BT}^{Dsp} \times SOC_{t - 1} $$

7 $$ {\mathrm{P}}_{{{\mathrm{BT}},{\mathrm{t}}}}^{{{\mathrm{Dch}}}} \times {\mathrm{P}}_{{{\mathrm{BT}},{\mathrm{t}}}}^{{{\mathrm{Ch}}}} = 0 $$

### H_2_ESS model

EL, HT, and FC are the three primary parts of H_2_ESS. EL turns on when there is enough wind and solar power, and the power generation system makes more electricity than is needed. It uses extra electricity to split water into H₂, which is then stored in HT. FC, on the other hand, turns on when the power output does not meet the electrical demand. It generates electricity to fulfill the electricity demand.

The H₂ storage has two main functions: to supply FC for power generation and to provide HVs with fuel. The minimum EL power (${P}_{min}^{EL}$) is defined in Eq. (8) by 10% of its maximum power (${P}_{max}^{EL}$)^24^.

8 $$ P_{\min }^{EL} = 0.1 \times P_{\max }^{EL} $$

The state of health of the hydrogen tank (${\mathrm{S}\mathrm{O}\mathrm{H}}_{\mathrm{t}}$) at time t is designated in Eq. (9), subject to the minimum SOH limit (${\mathrm{S}\mathrm{O}\mathrm{H}}_{\mathrm{m}\mathrm{i}\mathrm{n}}$) and maximum SOH limit (${\mathrm{S}\mathrm{O}\mathrm{H}}_{\mathrm{m}\mathrm{a}\mathrm{x}}$) of Eq. (10).

9 $$ SOH_{t} = SOH_{t - 1} + \frac{{N^{EL} \cdot P_{t}^{EL} \cdot \eta^{EL} \cdot \eta^{HT} \cdot \Delta t}}{{N^{HT} \cdot m_{r}^{HT} \cdot HHV}} - \frac{{N^{FC} \cdot P_{t}^{FC} \cdot \Delta t}}{{N^{HT} \cdot m_{r}^{HT} \cdot HHV \cdot \eta^{FC} \cdot \eta^{HT} }} - \frac{{E_{t}^{HV} }}{{N^{HT} \cdot m_{r}^{HT} \cdot HHV}} $$

10 $$ SOH_{\min } \le SOH_{t} \le SOH_{\max } $$

where ${m}_{r}^{HT}$ is the rated mass of the HT, and *HHV* is the high heating value of H₂. ${N}^{EL}$, ${N}^{FC}$, and ${N}^{HT}$ are the number of EL, FC, and HT with efficiencies of ${\eta }^{EL}$,${\eta }^{FC}$, and ${\eta }^{HT}$, respectively. The total energy consumed by the HVs at time *t*(${E}_{t}^{HV}$) is considered in the SOH calculation.

The H_2_ESS parameters considered in this paper are tabulated in Table 2.

### EV model

In this work, EVs are modeled as private cars considering the two modes of operation: G2V mode (EVs act as a load supplied by the MG) and V2G mode (EVs act as a source supplying the MG). Recent studies on smart energy layouts emphasize diverse paradigms for managing the widespread integration of EVs. For instance, a digital twin framework is investigated in^36^ to optimize the coordination and smart charging infrastructure of EV fleets. On the other hand, dynamic wireless charging methods integrated into smart grid infrastructure are examined in^37^ to support bidirectional power capabilities. While these large-scale or dynamic configurations often rely on a central aggregator to maintain grid stability, this study focuses on decentralized, residential-level energy exchange. Here, the household interface is supported by a localized programmable charger and a bi-directional meter to execute G2V and V2G cycles. Accordingly, the proposed model assumes that charging and discharging occur statically at home, while explicitly accounting for the stochastic behavior of individual private EVs represented by three random variables: daily driving distance (*d*), home arrival time (${t}_{a}$), and home departure time (${t}_{d}$).

The daily driving distance approximately follows a log-normal distribution, as depicted in Eq. (11). The departure and arrival times approximately follow a normal distribution, as illustrated in Eqs. (12) and (13)^24^. The mean values for driving distance, arrival, and departure time are denoted by ${\mu}_{d}^{EV}$,${\mu}_{ta}^{EV}$, and ${\mu}_{td}^{EV}$, respectively. ${\delta}_{d}$, ${\delta}_{{t}_{a}}$, and ${\delta}_{{t}_{d}}$ are the standard deviation*s* for driving distance, arrival, and departure times for EV, respectively.

11 $$ f\left( d \right) = \frac{1}{d}.\frac{1}{{\sqrt {2\pi } \delta_{d} }}exp\left( { - \frac{{\left( {lnd - \mu_{d}^{EV} } \right)^{2} }}{{2\delta_{d}^{2} }}} \right) $$

12 $$ f\left( {t_{d} } \right) = \frac{1}{{\sqrt {2\pi } \delta_{{t_{d} }} }}exp\left( { - \frac{{\left( {t_{d} - \mu_{td}^{EV} } \right)^{2} }}{{2\delta_{{t_{d} }}^{2} }}} \right) $$

13 $$ f\left( {t_{a} } \right) = \left\{ {\begin{array}{*{20}l} {\frac{1}{{\sqrt {2\pi } \delta_{{t_{a} }} }}exp\left( { - \frac{{\left( {t_{a} + 24 - \mu_{ta}^{EV} } \right)^{2} }}{{2\delta_{{t_{a} }}^{2} }}} \right)\quad 0 < t_{a} \le \left( {\mu_{ta}^{EV} - 12} \right)} \hfill \\ {\frac{1}{{\sqrt {2\pi } \delta_{{t_{a} }} }}exp\left( { - \frac{{\left( {t_{a} - \mu_{ta}^{EV} } \right)^{2} }}{{2\delta_{{t_{a} }}^{2} }}} \right)\quad \left( {\mu_{ta}^{EV} - 12} \right) < t_{a} \le 24} \hfill \\ \end{array} } \right. $$

**Fig. 6:**
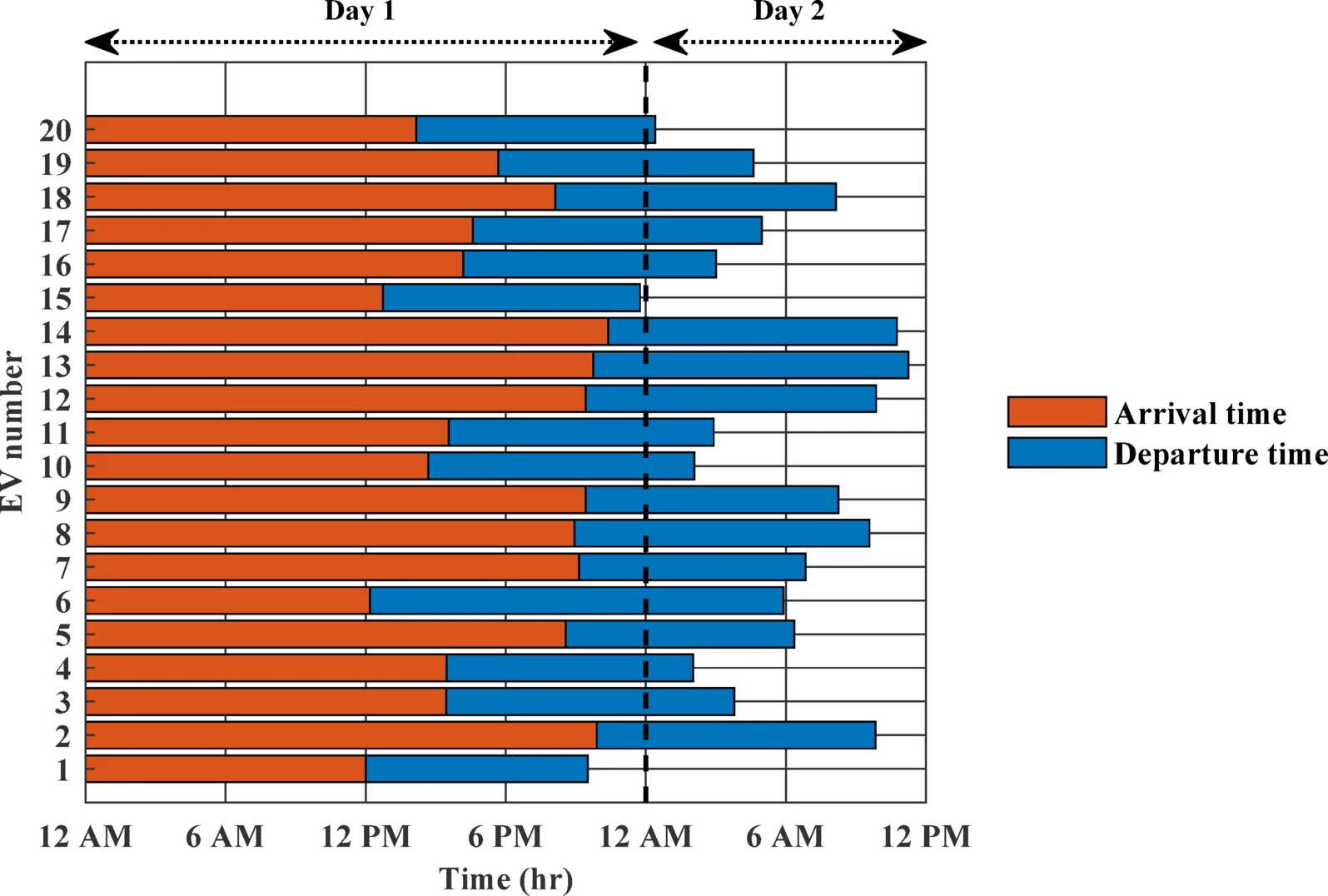
Arrival and departure times for the studied 20 EVs over 1.5 days as a sample.

For the investigated MG, which includes 20 EVs, the arrival and departure times are displayed in Fig. 6 over 1.5 days as a sample.

At home arrival time, ${\mathrm{S}\mathrm{O}\mathrm{C}}_{\mathrm{a}}$ represents the start of EV charging. The study uses the conventional approach of charging EVs slowly. Charging is performed by steadily adding power until the SOC reaches 100% at departure (${SOC}_{d}$). Equation (14) finds the SOC when the EV arrives home, where $\Delta {E}^{EV}$ is the energy consumption per km, and ${Q}^{EV}$ is the rated capacity of the EV in kWh.

14 $$ SOC_{a} = SOC_{d} - \frac{{d \cdot \Delta E^{EV} }}{{Q^{EV} }} $$

Deep discharge shortens the lifespan of EV batteries. If the remaining SOC is less than or equal to 0.2 when the EV arrives, it will charge until it reaches 1. If the SOC is above 0.2 when the owner gets home, he should focus on discharging schedules until it drops to 0.2. After that, it goes to charge scheduling until the SOC hits 1. Thus, the most important part of optimization is the timing of the charging and discharging. Accordingly, there are two situations:

- 1st: when the ${SOC}_{a}$ of an EV is less than 0.2, the EV charging time (${\mathrm{t}}_{\mathrm{c}\mathrm{h}1}$) will be according to Eq. (15).
- 2nd: when the ${SOC}_{a}$ of an EV is higher than 0.2, the discharging time (${\mathrm{t}}_{\mathrm{d}\mathrm{i}\mathrm{s}2}$) and charging time (${\mathrm{t}}_{\mathrm{c}\mathrm{h}2}$) are estimated as in Eq. (16).
  15 $$ t_{ch1} = Q^{EV} \cdot \frac{{\left( {1 - SOC_{a} } \right)}}{{P^{ch} /\eta^{EV} }} $$
  16 $$ \left\{ {\begin{array}{*{20}l} {t_{dis2} = Q^{EV} \cdot \frac{{\left( {SOC_{a} - 0.2} \right)}}{{P^{dis} /\eta^{EV} }}} \\ {t_{ch2} = Q^{EV} \cdot \frac{{\left( {1 - 0.2} \right)}}{{P^{ch} /\eta^{EV} }} } \\ \end{array} } \right. $$

where ${\eta }^{EV}$ describes the EV charging/discharging efficiency, and the charging and discharging power are denoted by ${P}^{ch}$ and ${P}^{dis}$, respectively^24^.

The total scheduled power of all EVs (${N}^{EV}$) at time *t* can be determined by summing the charging and discharging power of all EVs during the specified interval, as per Eqs. (17) and (18)^24^.

17 $$ P_{t}^{EV} = \mathop \sum \limits_{i = 1}^{{N^{EV} }} P^{ch} \cdot A_{i,t}^{EV} $$

18 $$ A_{i,t}^{EV} = \left\{ {\begin{array}{*{20}l} 1 \hfill & { If\, ith\, EV\, is\, charging \,at\, t} \hfill \\ 0 \hfill & {If\, ith\, EV\,is\, not \,charging\, or\, discharging\, at\, t} \hfill \\ { - 1} \hfill & {If \, ith \,EV \,is \,discharging \,at \,t} \hfill \\ \end{array} } \right. $$

where ${P}_{t}^{EV}$ is the total power for all EVs at time *t*; ${A}_{i,t}^{EV}$ is the ith EV status, whether charging or discharging at time *t*, and the charging and discharging powers are equal. If this total power is positive at any time, the EVs consume more power from the MG than they discharge, and vice versa.

### HV model

HVs are also modeled in this study as private cars, where the selected 10 HVs are Toyota Mirai. The HVs are incorporated within two modes: G2V mode, where the HVs purchase hydrogen from the MG, and V2G mode, in which HVs inject electrical power back into the MG, effectively acting as distributed energy sources. Charging and discharging HVs is done at a hydrogen station. Two uncertainties are considered in HV behavior: the arrival time at the station (${T}_{a}$), and the initial state of charge (${m}_{in}$) in the station. The arrival time and initial state of charge of the HV are modelled using the normal PDF as in Eq. (19)^22^.

19 $$ \left\{ {\begin{array}{*{20}l} {f\left( {T_{a} } \right) = \frac{1}{{\delta_{{T_{a} }} \sqrt {2\pi } }} \times \exp \left( { - \frac{{\left( {T_{a} - \mu_{{T_{a} }} } \right)^{2} }}{{2\delta_{{T_{a} }}^{2} }}} \right) } \hfill \\ {f\left( {m_{in} } \right) = \frac{1}{{\delta_{{m_{in} }} \sqrt {2\pi } }} \times \exp \left( { - \frac{{\left( {m_{in} - \mu_{{m_{in} }} } \right)^{2} }}{{2\delta_{{m_{in} }}^{2} }}} \right)} \hfill \\ \end{array} } \right. $$

where ${\mu}_{{T}_{a}}$ and ${\delta}_{{T}_{a}}$ represent the mean value and standard deviation of the arrival time, respectively. ${\mu}_{{m}_{in}}$ and ${\delta}_{{m}_{in}}$ represent the mean value and the standard deviation for the initial state of charge, respectively.

**Fig. 7:**
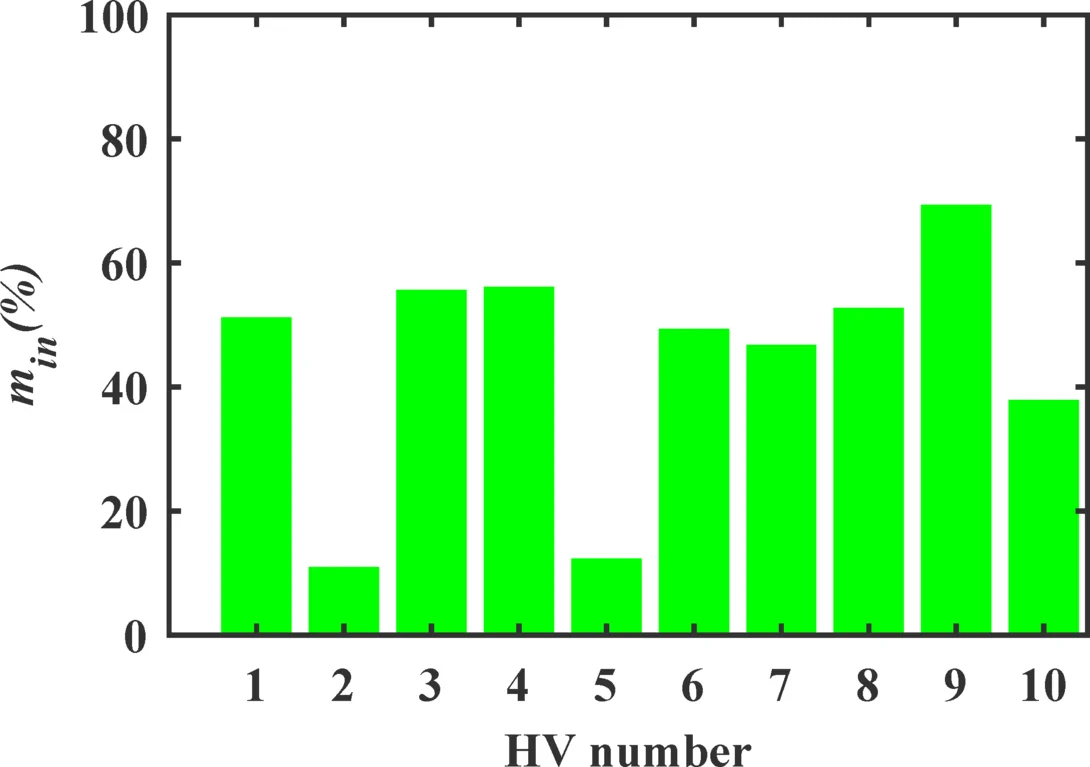
The initial state of charge of the 10 HVs over a sample day.

Figure 7 illustrates the initial state of charge of the 10 HVs in the investigated MG on a representative day, based on the selected values of ${\mu}_{{m}_{in}}$ and ${\delta}_{{m}_{in}}$. It is worth mentioning that the minimum and maximum values for the initial state of charging are selected as 10% and 90%, respectively. Based on the initial state of charging of the *ith* HV (${m}_{in}^{i}$); V2G or G2V mode is activated as follows:

- When ${m}_{in}^{i}$ is less than 30%, it will charge until 100%. The maximum energy stored in the HV (${E}_{max}^{HV}$) depends on its rated tank capacity (${m}^{HV}$) and efficiency in V2G mode (${\eta }^{HV}$), as given in Eq. (20).
  20 $$ E_{\max }^{HV} = m^{HV} \times HHV \times \eta^{HV} $$

(b) When ${m}_{in}^{i}$ exceeds 80%, it discharges power. The only limitation for HV is the discharge state. The limitation arises as the cooling capacity of the fuel cell system is restricted to 10% of the HV total capacity^38^. Consequently, Eq. (21) is employed to evaluate this limit, where ${P}_{max}^{HV,V2G}$ is the maximum power transferred from the HV to the MG.
  21 $$ P_{\max }^{HV,V2G} = 0.1 \times E_{\max }^{HV} $$

(c) Finally, when ${\mathrm{m}}_{\mathrm{i}\mathrm{n}}^{\mathrm{i}}$ is between 30 and 80%, the optimizer determines whether to charge or discharge.

In G2V mode, the total energy consumed by all HVs (${N}^{HV}$) at time *t* (${E}_{t}^{HV}$) is calculated using Eq. (22), while approximately 3 min are assumed for fast charging of each HV^39^.

22 $$ \left\{ {\begin{array}{*{20}l} {E_{t}^{HV} = \mathop \sum \limits_{i = 1}^{{N^{HV} }} \left( {\left( {100\% - m_{in}^{i} } \right) \times E_{\max }^{HV} } \right) \cdot A_{i,t}^{HV} } \hfill \\ { A_{i,t}^{HV} = \left\{ {\begin{array}{*{20}l} 1 \hfill & {If \,the\, ith \,HV \,is \,in \,G2V\, mode\, at\, time\, t} \hfill \\ 0 \hfill & {otherwise} \hfill \\ \end{array} } \right. } \hfill \\ \end{array} } \right. $$

In V2G mode, the cumulative power transferred from HVs to the MG at time *t* (${P}_{t}^{HV,V2G}$) is calculated as given in Eq. (23).

23 $$ \left\{ {\begin{array}{*{20}l} {P_{t}^{HV,V2G} = \mathop \sum \limits_{i = 1}^{{N^{HV} }} P_{\max }^{HV,V2G} \cdot U_{i,t}^{HV} } \hfill \\ { U_{i,t}^{HV} = \left\{ {\begin{array}{*{20}l} 1 \hfill & {If\, the\, ith\, HV\, is\, in\, V2G\, mode \, at\, time\, t} \hfill \\ 0 \hfill & {otherwise} \hfill \\ \end{array} } \right. } \hfill \\ \end{array} } \right. $$

The parameters used in this study for EVs and HVs are shown in Table 2*.*

## Problem formulation

**Fig. 8:**
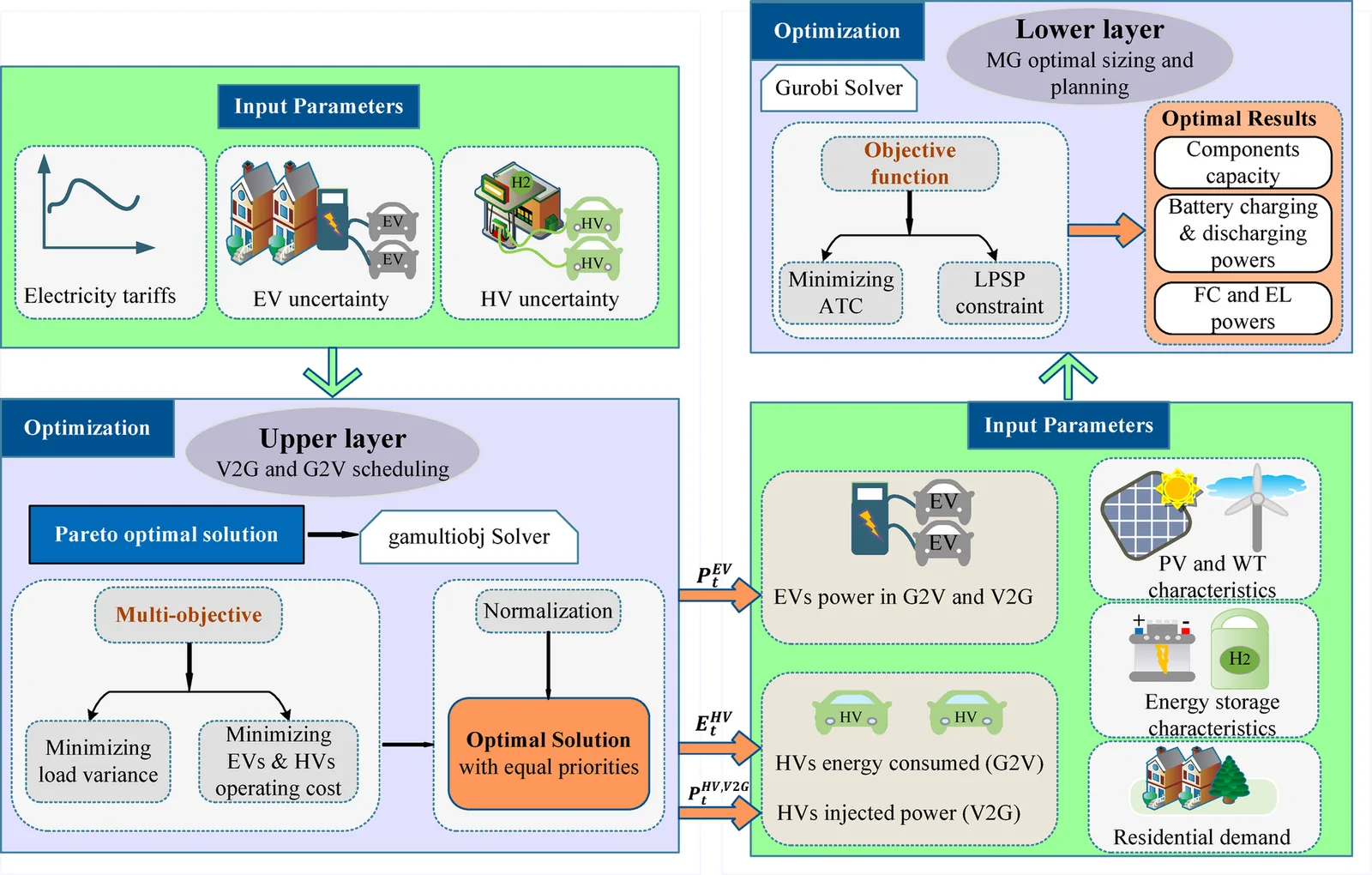
The proposed double-layer framework.

The proposed double-layer optimization model is displayed in Fig. 8, in which both upper and lower layers are multi-objective functions serving distinct but complementary stakeholder perspectives, as follows:

- The upper-layer model addresses the interests of both the MG operator and vehicle owners simultaneously through two competing objectives: minimizing the MG load variance (presenting the MG operator's perspective), while minimizing the total operational expenses for EV and HV owners (presenting the vehicle owners' perspective), as it reduces charging costs and maximizes V2G revenue. The conflict between these two stakeholders is reconciled through the Pareto front generated by NSGA-II, from which a compromise solution is selected via weighted aggregation. The resulting optimal V2G and G2V scheduling decision variables (${P}_{t}^{EV}$, ${E}_{t}^{HV}$, and ${P}_{t}^{HV,V2G}$) are passed to the lower layer as fixed coupling variables.
- The lower-layer model operates solely from the MG operator's perspective, targeting the minimization of both the ATC and the LPSP for optimal component sizing, solved using the Gurobi solver. It receives the coupling variables from the upper layer as fixed inputs, which reshape the net load profile seen by the MG. This coordinated vehicle behavior reduces the needed installed capacity for MG components, thereby minimizing MG life-cycle costs at this level.

**Fig. 9:**
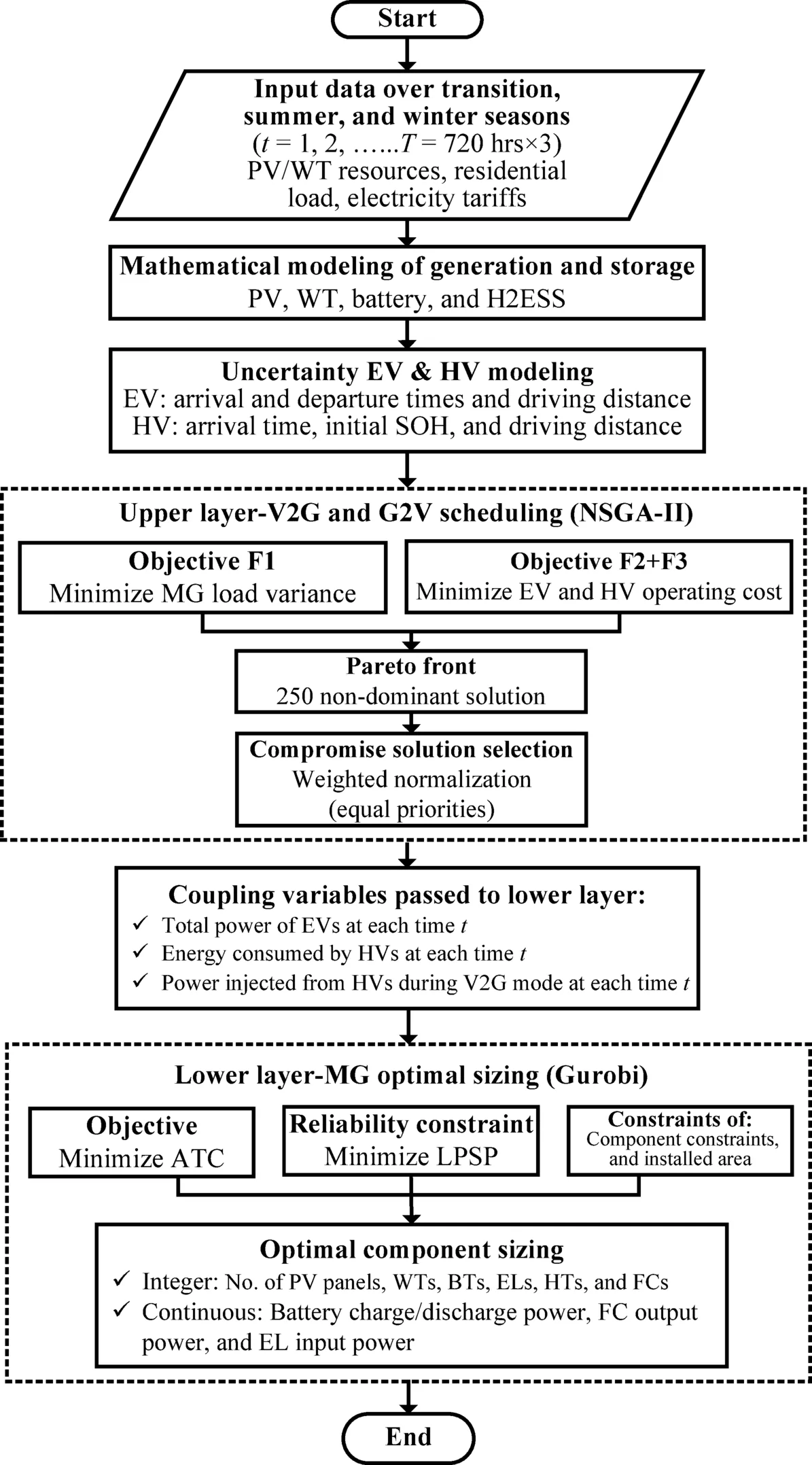
Flowchart of the proposed double-layer optimization methodology for the investigated MG under study.

The complete methodology flowchart of the proposed framework is illustrated in Fig. 9, summarizing the sequential data flow from stochastic uncertainty modelling through the upper-layer NSGA-II scheduling optimization, coupling variable transfer, and lower-layer Gurobi sizing optimization to the final optimal results.

The objective functions, system constraints, and component constraints will be presented in this section. To minimize computational burden and reduce the dimensionality of the optimization problem, three characteristic months are chosen to represent seasonal variations over the year. This ensures computational efficiency while capturing seasonal variations in natural conditions throughout the year.

### Upper- layer optimization model

The upper layer is a multi-objective optimization problem solved using NSGA-II (via MATLAB's gamultiobj), resulting in a set of Pareto-optimal solutions. NSGA-II is selected for this layer as it natively generates a complete Pareto front across competing objectives in a single run, directly embeds PDF-sampled stochastic scenarios for EV and HV behavioral uncertainty within the population fitness evaluation, and handles the non-convex, mixed-integer search space introduced by V2G scheduling. These characteristics preclude the direct application of exact mathematical programming solvers such as Gurobi for this sub-problem.

To extract a single compromise solution, a post-processing step is applied in which the objective functions are normalized and aggregated using weighting coefficients that represent decision-makers' preferences among the individual objectives. The solution with the minimum overall weighted score is selected as the final operating point.

The optimization parameters are set as follows: a population size of 200, 500 iterations, a 0.9 crossover probability, and a 0.1 mutation probability.

#### Upper-layer objective function

The upper-layer objectives are to minimize the MG's load variance and operating costs for EV and HV owners. The decision variables are the initial charging and discharging times for each EV and the status of each HV upon reaching the charging station, whether for charging or discharging. The total simulation time in hours (*T*) is for the three months.

Equation (24) displays how the total load ($${P}_{t}^{TL}$$) fulfills the electrical load demand ($${P}_{t}^{L}$$)*,* the total power for all EVs ($${P}_{t}^{EV})$$, and the power injected from the HVs during V2G mode ($${P}_{t}^{HV,V2G}$$). Accordingly, the load variance objective is displayed in Eq. (25), which should be minimized.

24 $$ P_{t}^{TL} = P_{t}^{L} + P_{t}^{EV} - P_{t}^{HV,V2G} $$

25 $$ f_{1} = \frac{1}{T} \cdot \mathop \sum \limits_{t = 1}^{T} \left( {P_{t}^{TL} - \frac{1}{T} \cdot \mathop \sum \limits_{t = 1}^{T} P_{t}^{TL} } \right)^{2} $$

The operating cost for EV owners, which is the second objective, is calculated as displayed in Eq. (26), where ${c}_{t}^{EV}$ is the EV charging and discharging tariffs. The time slots for charging and discharging of *i* th EV are denoted by $\Delta {\mathrm{t}}_{\mathrm{i},\mathrm{c}\mathrm{h}}$ and $\Delta {t}_{i,dis}$, respectively.

26 $$ f_{2} = \mathop \sum \limits_{i = 1}^{{N^{EV} }} \left( {\mathop \sum \limits_{{t \in T_{ch} }} c_{t}^{EV} \cdot P^{ch} \cdot \Delta t_{i,ch} + \mathop \sum \limits_{{t \in T_{dis} }} c_{t}^{EV} \cdot P^{dis} \cdot \Delta t_{i,dis} - C_{V2G}^{EV} } \right) $$

As shown, the car battery has to be drained to the MG system to activate V2G response in EVs. This means that the battery will eventually run out of power. The cost of EV battery degradation due to V2G operation (${C}_{V2G}^{EV}$) is expressed by Eq. (27), where the degradation is quantified as a cost^24^.

27 $$ \left\{ {\begin{array}{*{20}l} {C_{V2G}^{EV} = \mathop \sum \limits_{{t \in T_{dis} }} \left( {P^{dis} \cdot \tau } \right) } \hfill \\ {\tau = \frac{{c^{Bat} }}{{n \cdot Q^{EV} \cdot \left( {SOC^{max} - SOC^{min} } \right)}}} \hfill \\ \end{array} } \right. $$

where *τ * is the loss cost of a battery (per kWh) in V2G mode, ${c}^{Bat}$ is the battery cost for an EV, and *n* denotes the maximum count of cycles for an EV battery.

The third objective, which is the operating cost for HV owners, is described in Eq. (28). It accounts for the charging cost for the HV, which is considered from the stored hydrogen in MG, the discharging revenue due to the participation in the V2G approach, and finally the degradation cost of the fuel cell in V2G mode (${C}_{V2G}^{HV}$). It is worth noting that the FC stack accounts for approximately 70% of the total cost of the Toyota Mirai^40^.

28 $$ f_{3} = \mathop \sum \limits_{i = 1}^{{N^{HV} }} \left( {\mathop \sum \limits_{{t \in T_{ch} }} c_{t}^{HV} \cdot m_{t}^{ch} - \mathop \sum \limits_{{t \in T_{dis} }} c_{t}^{HV} \cdot m_{t}^{dis} + C_{V2G}^{HV} } \right) $$

where ${c}_{t}^{HV}$ is the HV charging and discharging tariffs at time *t*. ${m}_{t}^{ch}$ and ${m}_{t}^{dis}$ are the charging and discharging masses at time *t*, as presented in Eqs. (29) and (30).

29 $$ m_{t}^{ch} = \left( {1 - m_{in}^{ HV} } \right) \times m^{HV} $$

30 $$ m_{t}^{dis} = \frac{{P_{\max }^{HV,V2G} \cdot \Delta t_{i,dis} }}{HHV } $$

For the FC replacement cost of ${\mathrm{c}}^{\mathrm{F}\mathrm{C}}$, and the energy consumption for HV per km of ${\Delta E}^{HV}$, the degradation cost of the FC in V2G mode is estimated as shown in Eq. (31), where the maximum driving distance for the Toyota Mirai car during its warranty (${d}_{max}^{HV}$*)* is almost the distance after which the FC should be replaced.

31 $$ C_{V2G}^{HV} = \frac{{P_{\max }^{HV,V2G} \cdot \Delta t_{i,dis} }}{{\Delta E^{HV} \cdot d_{\max }^{HV} }} \cdot c^{FC} $$

The charging and discharging tariffs for EVs and HVs are depicted in Table 3, whereas the electrolyzer requires 50 kWh (including its efficiency) to produce 1 kg of hydrogen^41^.

**Table 3:** Charging and discharging tariffs for EVs and HVs^24^.

	Period	$${\mathrm{c}}_{\mathrm{t}}^{\mathrm{E}\mathrm{V}}$$($/kWh)	$${\mathrm{c}}_{\mathrm{t}}^{\mathrm{H}\mathrm{V}}$$($/kg)
Peak	17:00–22:00	0.12	6
Normal	8:00–17:00 22:00–24:00	0.097	4.85
Valley	0:00–8:00	0.051	2.55

The three objective functions are normalized to eliminate the influence of differing units and scales, thereby ensuring a consistent and comparable assessment of the solutions. To appraise the set of Pareto-optimal solutions within the multi-objective optimization framework, a composite objective function (*f*) is constructed using weighting coefficients (${\omega}_{j}$) as shown in Eq. (32).

32 $$ {\text{min }}f = { }\mathop \sum \limits_{j = 1}^{3} \omega_{j} \left( {\frac{{f_{j} - f_{j}^{{{\mathrm{min}}}} }}{{f_{j}^{{{\mathrm{max}}}} - f_{j}^{{{\mathrm{min}}}} }}} \right) $$

where *j* represents the objective number, ${f}_{j}^{min}$, and ${f}_{j}^{max}$ are the minimum and maximum values for the individual objective *j*, respectively.

It should be noted that selecting weighting coefficients represents decision-makers' preferences among the three objectives. In this study, equal weighting is adopted for all objectives with ${\omega}_{j}=1/$ 3, satisfying the condition of $\sum_{j=1}^{3}{\omega}_{j}=1$.

#### Upper-layer constraints

1. EV charging and discharging time constraints: Each EV has to follow a set of rules to ensure it works with the real world and is safe to charge and discharge. According to Eq. (33), where both the initial discharging time ($${t}_{dis}^{ini}$$) and the initial charging time ($${t}_{ch}^{ini}$$) should fall between the arrival ($${t}_{a})$$ and departure ($${t}_{d}$$) times.
  33 $$ \left\{ {\begin{array}{*{20}l} {t_{a} < t_{dis}^{ini} < t_{d} } \hfill \\ {t_{a} < t_{ch}^{ini} < t_{d} } \hfill \\ \end{array} } \right. $$

(2) EV battery's SOC constraints: The SOC of the EV battery should follow Eq. (34) when $SO{C}_{a}>0.2$ and Eq. (35) when $SO{C}_{a}\le 0.2$.
  34 $$ SOC_{min} \le SOC \le SOC_{max} $$
  35 $$ SOC_{a} \le SOC \le SOC_{max} $$

### Lower-layer optimization model

This lower layer is solved using the Gurobi Optimizer interfaced through MATLAB. Gurobi was selected due to its robustness and computational efficiency in solving large-scale mixed-integer quadratically constrained programming problems, particularly those involving nonlinear quadratic objective functions and constraints commonly encountered in energy management and microgrid optimization applications.

#### Lower-layer objective function

The objective function is to minimize the MG model's ATC, which includes the capital investment cost (*CI*),operation and maintenance cost (*OM*), and replacement cost (*RC*) of the system's parts (PV, WT, BT, FC, EL, HT),but excluding EV and HV purchasing costs, as illustrated in Eq. (36).

36 $$ ATC = C_{CI, OM}^{PV} + C_{CI, OM}^{WT} + C_{CI, OM,RC}^{BT} + C_{CI, OM,RC}^{FC} + C_{CI, OM,RC}^{EL} + C_{CI, OM}^{HT} $$

The project lifespan (*N*) and the interest rate ($\mathrm{i}\mathrm{r}$) are assumed to be 20 years and 10%, respectively. Accordingly, the capital recovery factor (CRF) is calculated as given in Eq. (37)^22^. The components of battery, FC, and EL have *RC*, since their lifespans are shorter than the project lifetime. The *RC* of each component equals the initial *CI*. To convert the *RC* to its present value, the discount factor *σ (u)* is applied, where *u* denotes the replacement year, as expressed in Eq. (38)^22^.

37 $$ CRF\left( N \right) = \frac{{ir(1 + ir)^{N} }}{{(1 + ir)^{N} - 1}} $$

38 $$ \sigma \left( u \right) = \frac{1}{{\left( {1 + ir} \right)^{u} }} $$

Consequently, the terms of Eq. (36) are calculated as shown in Eq. (39):

39 $$ \begin{gathered} C_{CI, OM}^{PV} = CRF\left( {20} \right) \cdot P_{PV,r} \cdot N^{PV} \cdot \left( {C_{PV}^{CI} + C_{PV}^{OM} } \right) \hfill \\ C_{CI, OM}^{WT} = CRF\left( {20} \right) \cdot P_{WT,r} \cdot N^{WT} \cdot \left( {C_{WT}^{CI} + C_{WT}^{OM} } \right) \hfill \\ C_{CI, OM,RC}^{BT} = CRF\left( 5 \right) \cdot N^{BT} \cdot (C_{BT}^{CI} + C_{BT}^{OM} + C_{BT}^{OM} \cdot \mathop \sum \limits_{u = 5,10,15} \sigma \left( u \right)) \hfill \\ C_{CI, OM,RC}^{FC} = CRF\left( 5 \right) \cdot P_{\max }^{FC} \cdot N^{FC} \cdot \left( {C_{FC}^{CI} + C_{FC}^{OM} + C_{FC}^{OM} .\mathop \sum \limits_{u = 5,10,15} \sigma \left( u \right)} \right) \hfill \\ C_{CI, OM,RC}^{EL} = CRF\left( {10} \right) \cdot P_{\max }^{EL} \cdot N^{EL} \cdot \left( {C_{EL}^{CI} + C_{EL}^{OM} + C_{EL}^{OM} \cdot \sigma \left( {10} \right)} \right) \hfill \\ C_{CI, OM}^{HT} = CRF\left( {10} \right) \cdot m_{r}^{HT} \cdot N^{HT} \cdot \left( {C_{HT}^{CI} + C_{HT}^{OM} } \right) \hfill \\ \end{gathered} $$

Thus, the decision variables for the lower optimization model will be:

1. The number of WTs, PV panels, BTs, ELs, HTs, and FCs.
2. Charging and discharging power for batteries ∀t ∈ T.
3. Battery state of charge (SOC) ∀t ∈ T.
4. Fuel cell power ∀t ∈ T.
5. Electrolyzer power ∀t ∈ T.
6. Hydrogen tank state of health (SOH) ∀t ∈ T.
7. Unmet power by the MG ∀t ∈ T.

#### Lower-layer constraints

The operation of the MG is governed by a set of system constraints that must be enforced to guarantee reliable performance and attain the optimal sizing solution. Therefore, in addition to Eqs. (7) and (8), the following constraints should be considered:

1. Power balance constraint:
  40 $${N}^{PV}.{P}_{t}^{PV}+{N}^{WT}.{P}_{t}^{WT}+{N}^{BT}.\left({P}_{t}^{BT,ch}-{P}_{t}^{BT,dis}\right)+{N}^{FC}.{P}_{t}^{FC}-{N}^{EL}.{P}_{t}^{EL}-{P}_{t}^{TL}+{P}_{t}^{lost }=0$$

where ${P}_{t}^{lost}$ is the unmet load power at time *t* to avoid oversizing of the system components. The critical load is assumed to represent 20% of the total load power in this study.

(2) Reliability constraint:

Using the ε-constraint method, the reliability objective is converted into a constraint. LPSP is adopted as the reliability index, as defined in Eq. (41).

41 $$ \mathop \sum \limits_{t = 1}^{T} P_{t}^{lost } \le LPSP\mathop \sum \limits_{t = 1}^{T} P_{t}^{L} $$

(3) Component constraints (number & size):
  42 $$ N_{min}^{j} \le N^{j} \le N_{max}^{j} $$
  43 $$ P_{min}^{j} \le P_{t}^{j} \le P_{max}^{j} $$

where ${\mathrm{P}}_{\mathrm{m}\mathrm{i}\mathrm{n}}^{\mathrm{j}}$ and ${\mathrm{P}}_{\mathrm{m}\mathrm{a}\mathrm{x}}^{\mathrm{j}}$ are the minimum and maximum output power limits for component $\mathrm{j}$, respectively. ${\mathrm{N}}_{\mathrm{m}\mathrm{i}\mathrm{n}}^{\mathrm{j}}$ and ${\mathrm{N}}_{\mathrm{m}\mathrm{a}\mathrm{x}}^{\mathrm{j}}$ are the minimum and maximum numbers of each component $\mathrm{j}$, respectively.

(4) Installed area constraint:
  44 $$ {\mathrm{N}}^{{{\mathrm{PV}}}} \cdot {\mathrm{A}}^{{{\mathrm{PV}}}} + {\mathrm{N}}^{{{\mathrm{WT}}}} \cdot {\mathrm{A}}^{{{\mathrm{WT}}}} \le {\mathrm{A}}^{{{\mathrm{MG}}}} $$

where ${A}^{PV}$ and ${A}^{WT}$ are the required areas of a PV panel and a WT, respectively, and the total space for the MG (${A}^{MG}$) is 20,000 m^2^. The WTs should be spaced 8D apart in the downwind direction and 5D apart in the crosswind direction to capture the maximum power of the WT system^42^, where D is the WT rotor diameter.

## Results and discussion

This section investigates how operational conditions affect the optimal sizing of MG components. The detailed parameters of all system components (PV, WT, BT, EL, FC, HT, EVs, and HVs) are listed in Table 2. The charging and discharging tariffs for EVs and HVs are summarized in Table 3. Building on these parameters, the investigations are presented across two main case studies. For such case studies, the optimal sizing of the MG components is determined across examined scenarios, after which the best-performing scenario is studied in greater detail. The sequence for the carried out analyses is summarized as follows:

- First, case study 1 is investigated, where no unmet load power is permitted; five scenarios (S1-S5) that combine G2V and V2G integration for EVs, HVs, or both are evaluated.
- Second, the best-performing scenario, S5, is then analyzed further: the optimized EV and HV scheduling profiles over a 48-h horizon are presented, and the power-sharing dynamics are examined among all MG components over a representative day.
- Third, the analysis is then extended to the second case study by applying variable LPSP values (0–0.2) to S5.

### Case study 1 with no unmet load power

Several scenarios, summarized in Table 4, are investigated to evaluate the influence of EVs and HVs on the optimal sizing of rural MGs. The five scenarios consider different combinations of G2V and V2G integration for EVs, HVs, or both. In these scenarios, there would be no unmet load power. The optimal results for all examined scenarios are summarized in Table 5, with the key findings outlined as follows:

- Without any EVs or HVs in the MG in S1 (base scenario), the minimum cost for MG sizing is $238,579.4. But the emissions in this scenario would increase significantly due to the use of conventional diesel vehicles.
- Integrating both EVs and HVs in G2V-only mode in S2 raises the total cost by 46.9% relative to S1, reaching $350,424.5.
- A lower cost is achieved when applying V2G with HVs only (S4) compared to S3 (which enables V2G for EVs only). It is agreed that HV-based V2G operation is more effective due to the higher cost of hydrogen charging and discharging compared to electrical power. However, it is expected to be reduced considerably in the future.
- When both EVs and HVs are integrated using both G2V and V2G modes in S5, a total cost of $308,141.9 is attained, corresponding to cost reductions of 8.35% and 3.75% compared to S3 and S4, respectively.
- The optimization framework tends to limit the expansion of PV capacity due to the associated increase in battery storage costs required to manage peak generation. Instead, the WT system, which provides a stable and continuous power output, is slightly increased to serve as a reliable baseline generation source. This trade-off is clear in scenarios S4 and S5, where a moderate increase in WT system capacity helps balance system reliability while avoiding excessive investment in storage-intensive PV generation.

**Table 4:** Scenarios of EVs and HVs for case study 1.

Scenario		G2V for EV	V2G for EV	G2V for HV	V2G for HV
S1	No EVs or HVs	×	×	×	×
S2	With EVs and HVs	✓	×	✓	×
S3		✓	✓	✓	×
S4		✓	×	✓	✓
S5		✓	✓	✓	✓

**Table 5:** Optimal planning results for the investigated scenarios in case study 1.

	$${N}^{PV}$$	$${N}^{WT}$$	$${N}^{BT}$$	$${N}^{FC}$$	$${N}^{EL}$$	$${N}^{HT}$$	ATC ($)
S1	873	1	701	16	52	221	238,579.4
S2	1234	0	1093	19	88	162	350,424.5
S3	1153	2	1057	18	82	171	336,229.7
S4	1095	7	984	23	67	187	320,145.0
S5	993	8	942	29	56	201	308,141.9

As shown in Table 5, the best scenario among the examined scenarios, including EVs and HVs, is S5. It achieves the minimum cost in an emission-free MG. Therefore, S5 is the scenario applied to analyze the system behavior.

#### Results for integrating G2V & V2G for EVs and HVs (S5)

**Fig. 10:**
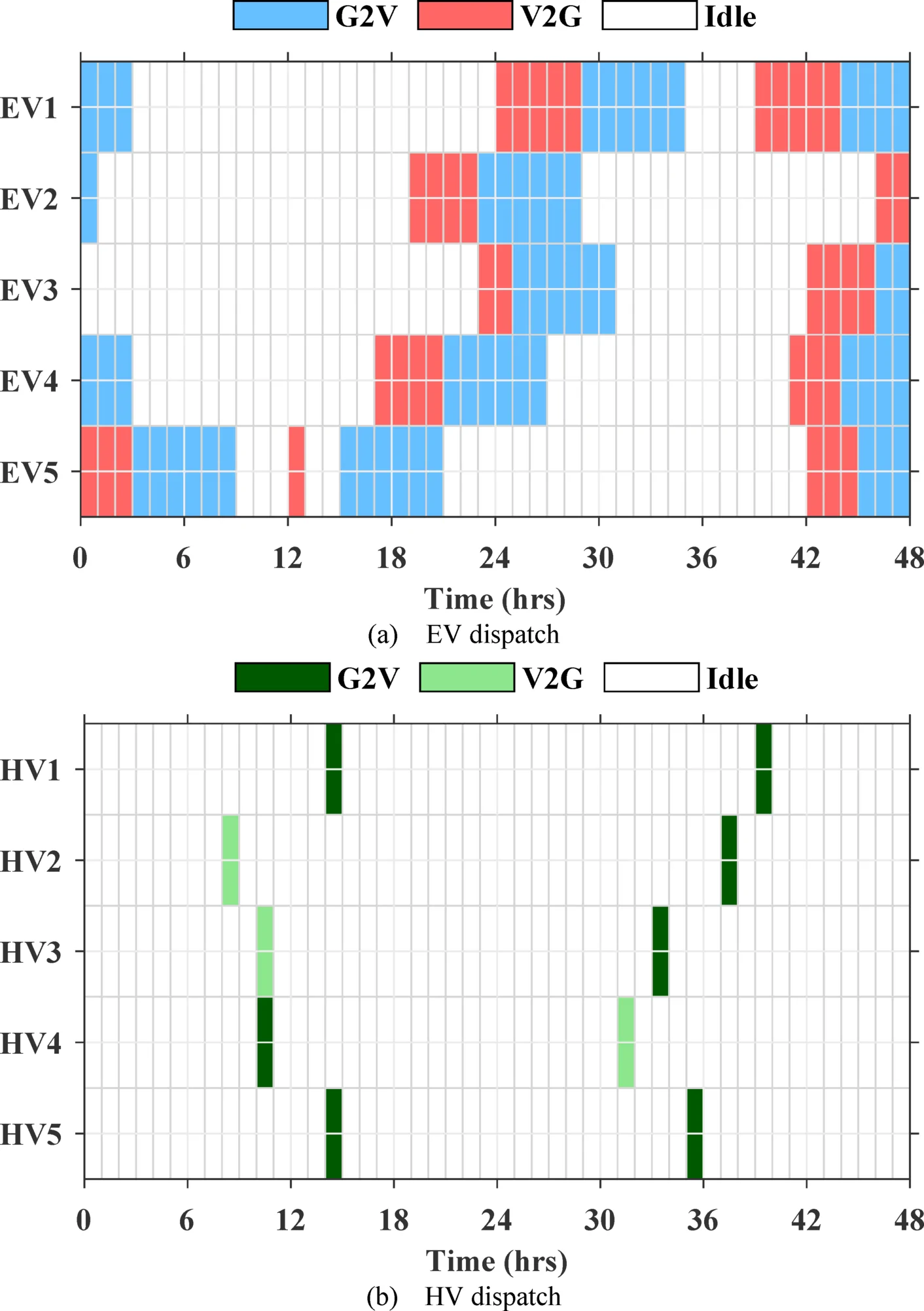
Optimized scheduling profiles for two consecutive days.

For S5, which considers both G2V and V2G integration for EVs and HVs, the optimized scheduling profiles for the EV and HV fleets over a continuous 48-h horizon are illustrated in Fig. 10a b, respectively. To evaluate the operational behavior of the proposed hierarchical framework, representative samples from five EVs and five HVs are analyzed, with their states reindexed to a 0–48-h temporal scale for clarity.

As illustrated in Fig. 10a, the framework effectively coordinates the G2V charging and V2G discharging cycles of EVs. Similarly, Fig. 10b illustrates the dispatching schedule for HVs when arriving at the charging station, in which the algorithm manages charging and discharging events based on the vehicles' initial state of charge and MG requirements. The proposed framework strategically prioritizes V2G discharge events for both vehicle fleets during peak-demand intervals to enhance MG flexibility. Conversely, vehicle charging is intelligently scheduled during off-peak periods to minimize load variance and reduce operational expenses.

**Fig. 11:**
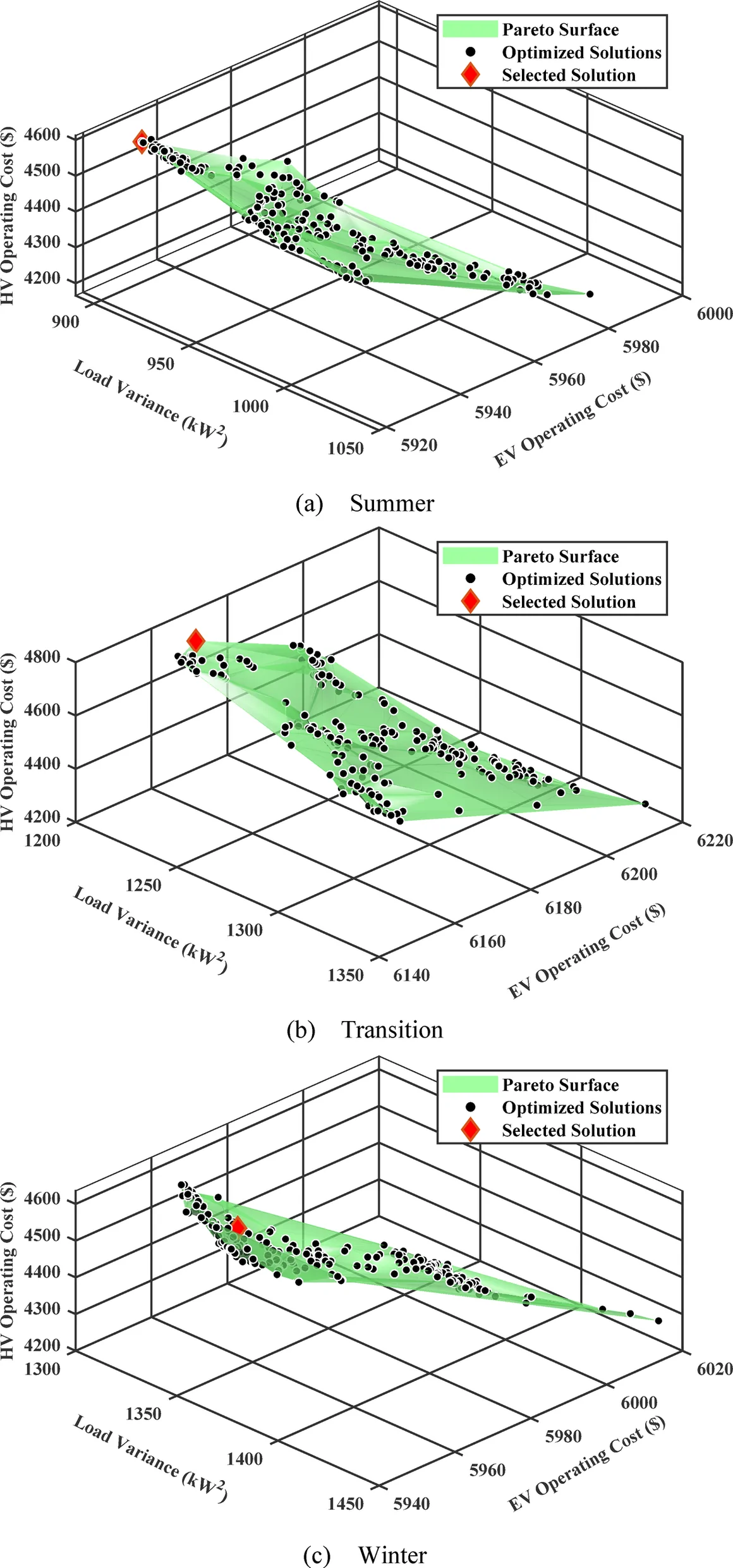
Pareto optimal fronts for the upper layer in the three seasons.

Figure 11 shows the 3D Pareto optimal fronts for the upper optimization layer. These fronts show the trade-offs between load variance, EV operating costs, and HV operating costs across the three seasons. Quantitatively, the selected compromise solution (achieved by equal weights for the three objectives) is highlighted by the red diamond in Fig. 11a, b, and c. It demonstrates the framework's efficacy in balancing these three conflicting objectives.

The optimized costs of running an EV are $5,935.9 in the summer, $6,168.3 in the transition month, and $5,960.1 in the winter. Concurrently, the HV operating expenses are effectively minimized to $4,512.8, $4,722.5, and $4,591.5 for the corresponding months, respectively.

These results reflect the successful optimization of the second and third objectives, in which HV costs are minimized by leveraging off-peak windows shortly after arrival at the charging station, even when their contributions to evening peak shaving are limited by their operational schedules.

**Fig. 12:**
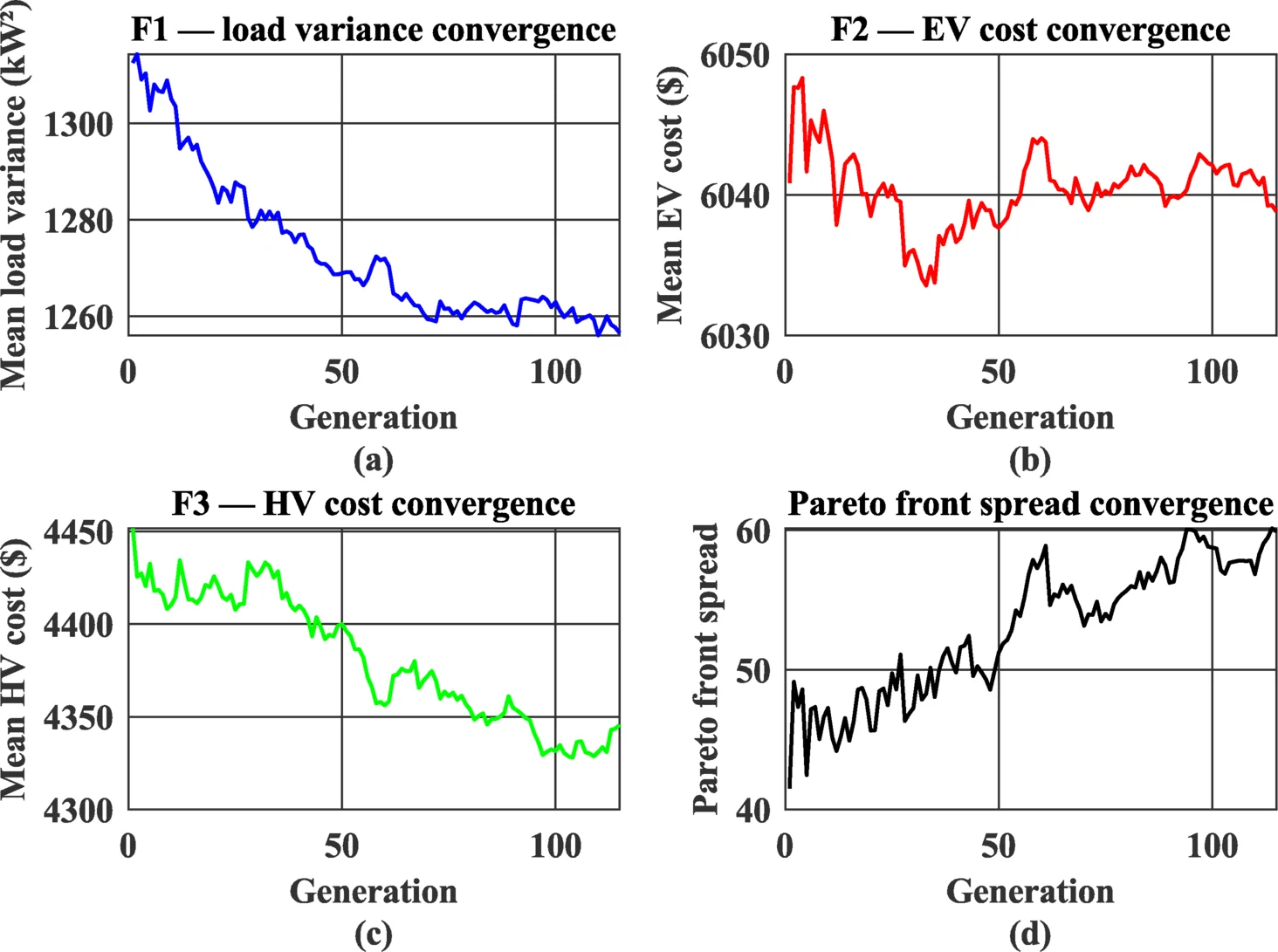
Convergence behavior of the NSGA-II upper-layer optimizer for the transition month (representative case): (**a**) mean load variance (F1), (**b**) mean EV operational cost (F2), (**c**) mean HV operational cost (F3), and (**d**) Pareto front spread across generations.

The convergence behavior of the NSGA-II upper-layer optimizer is evaluated independently for each representative month, and the results are summarized in Table 6. Figure 12 presents the convergence curves for the transition month as a representative case; all three objective functions exhibit clear, consistent downward trends with no divergence. F1 (load variance) achieves the most significant reduction across all months (2.94–4.33%), while F2 (EV cost) and F3 (HV cost) show improvements of 0.15–0.20% and 1.77–2.62%, respectively. The Pareto front spread increases consistently from 40–43 to 49–59 across all runs, confirming that population diversity is maintained throughout, yielding 250 well-distributed non-dominated solutions per run. All runs terminate due to the Function Tolerance criterion, confirming mathematically sound convergence. The total computational time per month ranges from 12.31 to 17.40 min, plus approximately 45 min for the upper-level optimization, using an Intel Core i7 processor with 16 GB RAM.

**Table 6:** NSGA-II convergence performance metrics across the three representative months.

Metric	Summer	Transition	Winter
Generations completed	118	116	102
Function evaluations	59,000	58,000	51,000
Computational time (min)	17.4	15.27	12.31
Pareto front size	250	250	250
F1 initial → final (kW^2^)	1430 → 1388	1315 → 1258	1023 → 985
F2 initial → final ($)	5994 → 5985	6048 → 6039	6022 → 6010
F3 initial → final ($)	4575 → 4455	4450 → 4345	4398 → 4320
Pareto spread initial → final	43 → 49	43 → 59	40 → 59

**Fig. 13:**
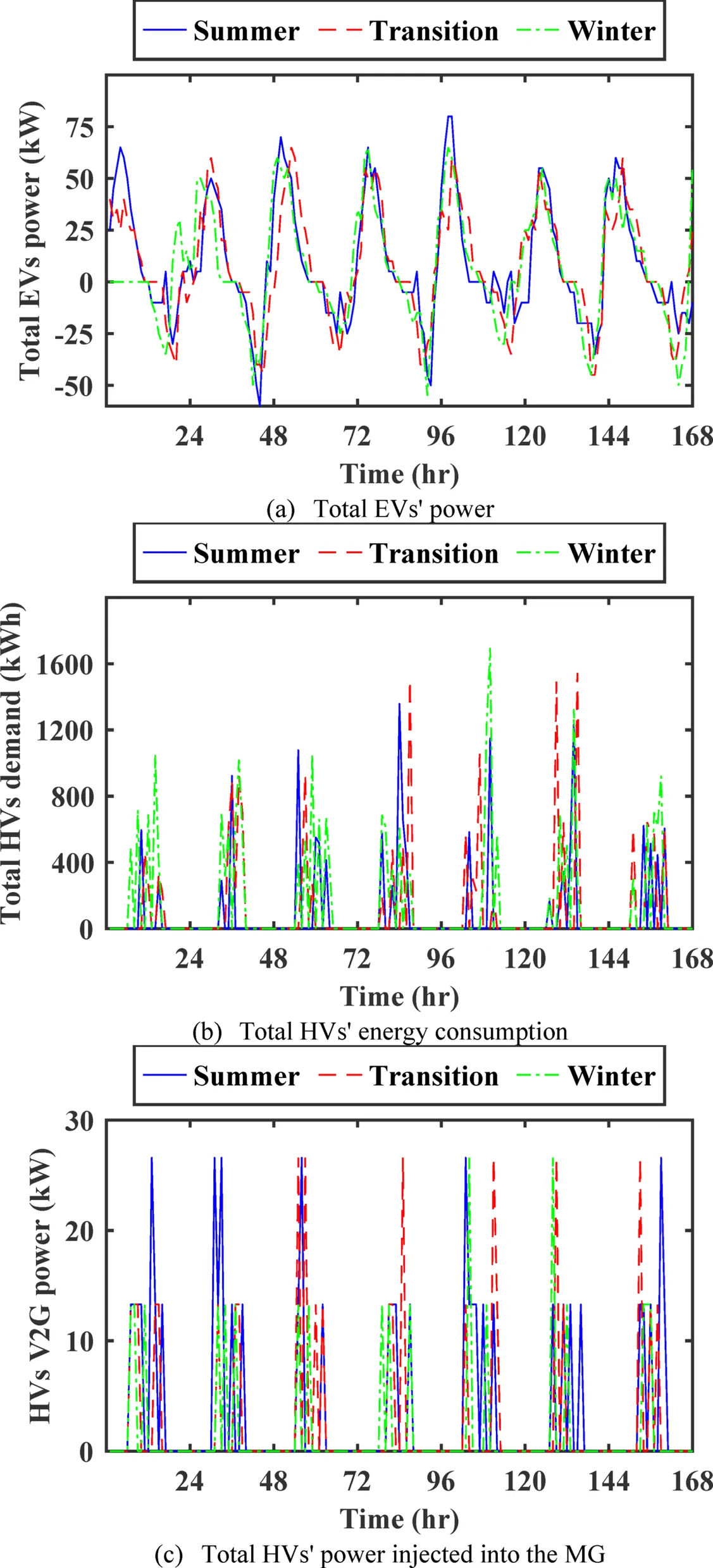
Output of the upper optimization layer for S5 during the first week in the three seasons.

The output of the upper optimization layer for S5 during the first week of the three seasons (168 h) is illustrated in Fig. 13. It encompasses the aggregated EV charging power, total HV energy consumption, and total HVs' power fed into the grid through V2G operation.

The total EV power shown in Fig. 13a includes both positive and negative values, indicating bidirectional EV operation. Positive values correspond to charging (G2V), where the aggregated charging power exceeds the discharging, while negative values indicate discharging (V2G), where EVs inject power back into the grid. The figure shows that charging and discharging events are distributed throughout the day rather than confined to fixed time intervals, reflecting a dynamic scheduling strategy. In general, charging tends to occur during low-demand periods, contributing to valley filling, whereas discharging is more frequent during higher-demand periods, supporting peak shaving. These patterns vary from day to day and across seasons, highlighting the adaptive role of EVs in responding to real-time grid conditions rather than strictly following predefined time windows.

Figure 13b depicts the energy required to operate HVs throughout the first week in summer, winter, and transition seasons. HV charging at the station is scheduled based on their arrival times and prevailing energy costs. The energy peaks occur at different times across days and seasons, indicating that energy consumption is not concentrated in a single period. Instead, HV refueling is dynamically scheduled based on vehicle usage patterns and operational conditions.

Figure 13c illustrates the power flow from HVs back to the grid in V2G mode. The highest power injection (V2G) typically occurs when the HVs are at the station. In V2G operation, hydrogen fuel cell vehicles inject electrical power into the grid rather than hydrogen because the electrical grid can only interface with electrical energy. The FC must first convert the hydrogen stored on board into electricity. Direct hydrogen injection is not feasible due to the absence of hydrogen infrastructure at grid nodes and the safety risks associated with handling high-pressure hydrogen. As a result, HVs operate as distributed generators in V2G mode, injecting power into the grid and improving its flexibility and reliability.

**Fig. 14:**
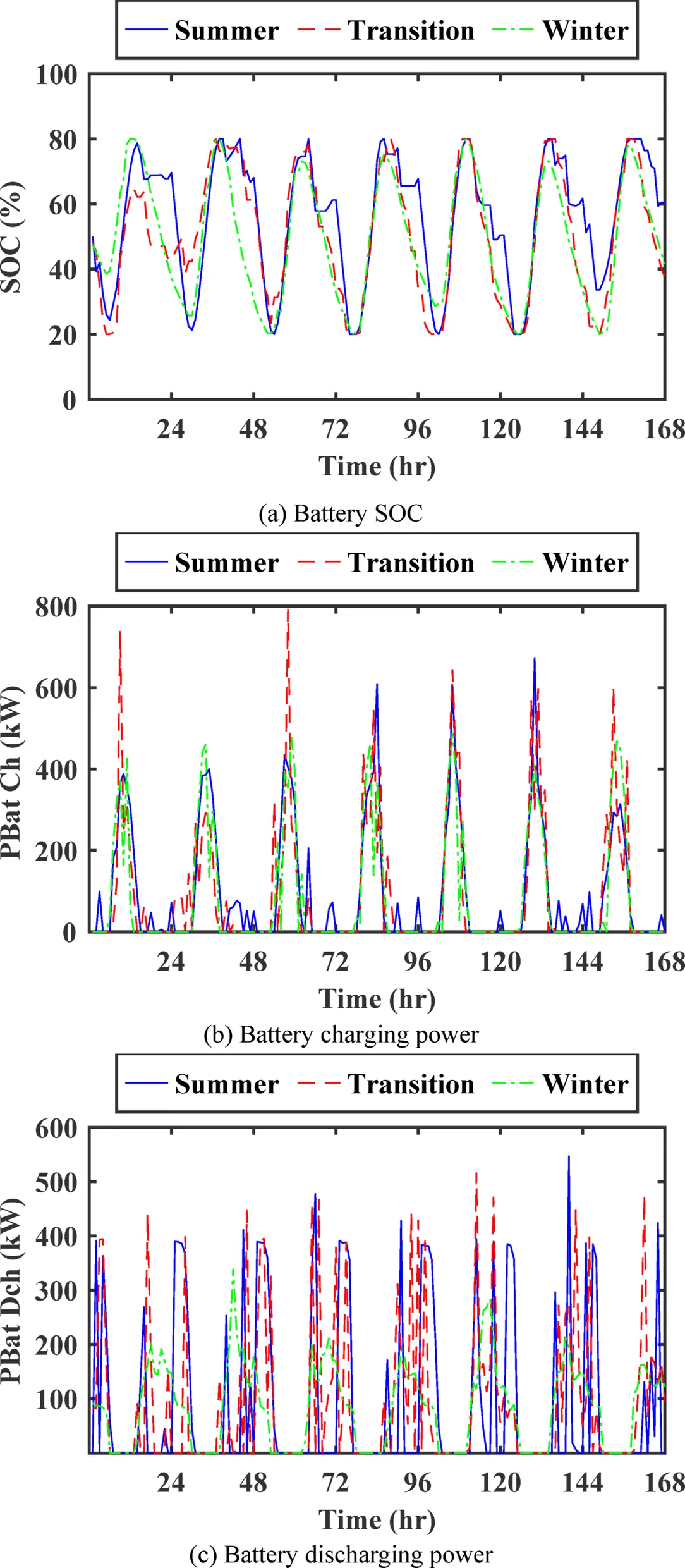
Battery system output for S5 during the first week in the three seasons.

The following analysis focuses on battery behavior in S5, identified as the optimal scenario. Figure 14a shows the battery SOC during the first week in the three seasons. The maximum SOC is typically reached around midday, coinciding with the absorption of surplus power generated by the PV system. Seasonal differences are not pronounced in SOC behavior, implying that short-term operational conditions and load-generation balance are more determinant for SOC behavior than seasonal variation, notwithstanding slight fluctuations in peak and minimum values.

Figure 14b illustrates the battery charging power during the first week in the three seasons. The battery charging period occurs when the MG has surplus power generation from the renewable sources. Consistent with SOC behavior, seasonal differences in charging peaks and patterns are not systematically significant, as both fluctuate considerably depending on daily operational conditions. Figure 14c shows the battery discharging power delivered to the MG during periods of insufficient renewable energy output. The discharge is most prominent during the night to meet the load after the sun sets. The least discharging power occurs during the winter season due to the small mismatches between generation and load.

**Fig. 15:**
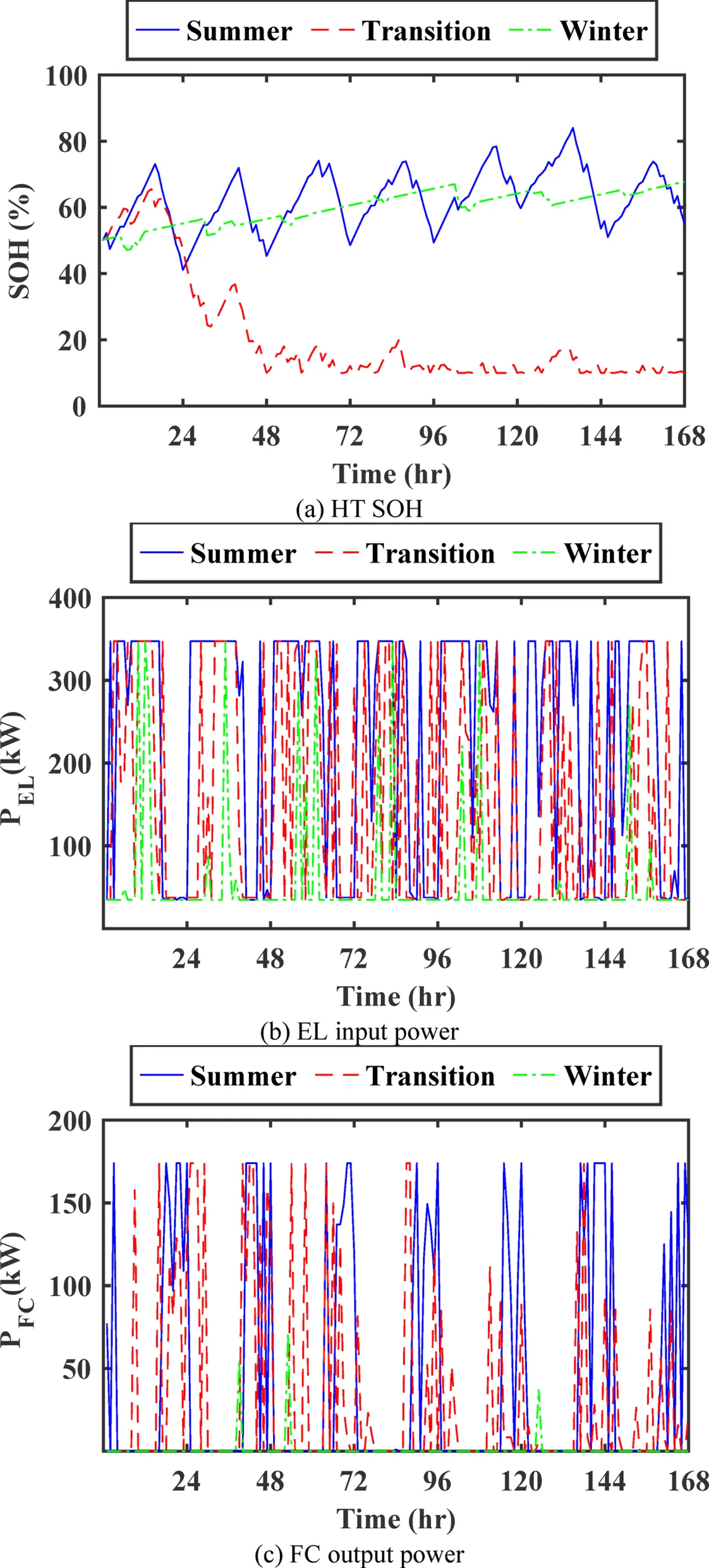
H_2_ESS output for S5 during the first week in the three seasons.

H_2_ESS output, including HT SOH, EL input power, and FC output power during the first week of the three seasons, is shown in Fig. 15. As shown in Fig. 15a, this operational mode explains the intensive cycling for the SOH in summer, indicating frequent charging and discharging cycles driven by active operation of both the EL and FC. In contrast, the winter profile exhibits a gradual increase in SOH with only minor fluctuations due to limited FC usage and the dominance of hydrogen production. During the transition season, the SOH rapidly declines and remains around the lower operational limit (10%), indicating continuous utilization of the hydrogen storage system. The high-frequency switching observed in EL and FC power outputs (Fig. 15c) indicates that the hydrogen system primarily facilitates sub-hourly power balancing of PV/WT and battery resources, rather than operating exclusively as a slow seasonal storage medium.

#### A detailed day analysis for S5

**Fig. 16:**
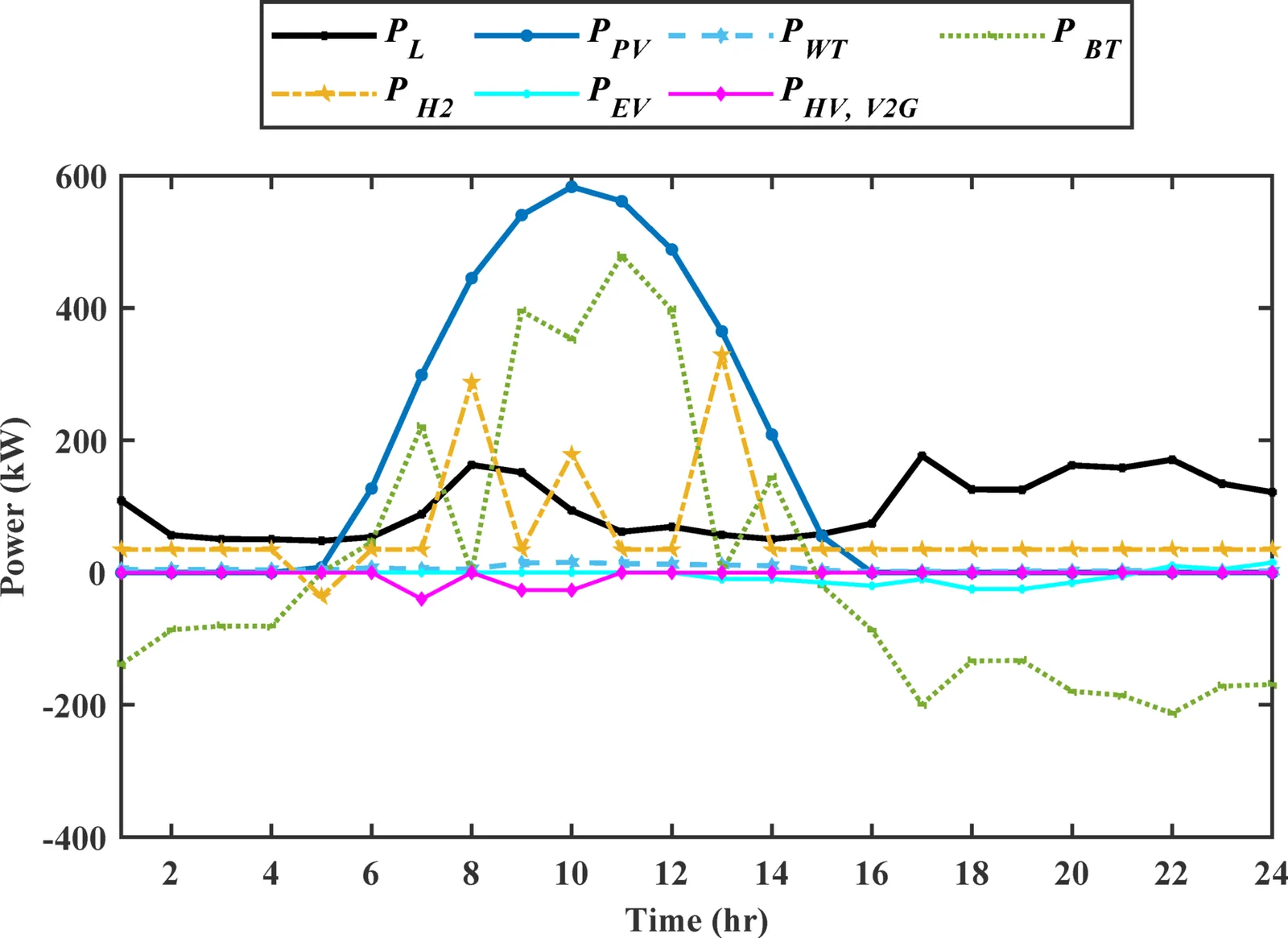
A sample of daily output power for MG components.

As a detailed example, Fig. 16 illustrates the power-sharing dynamics among all components of the MG over a representative operational day for scenario S5, as follows:

- The main source of power is PV generation. It has a bell-shaped profile (${P}_{PV}$), with peak output reaching 580 kW around noon (10:00–12:00), which is much higher than the instantaneous load demand (${P}_{L}$) during peak solar irradiance periods.
- The battery system (${P}_{BT}$) operates in discharge mode during periods of diminished solar availability, specifically from 0:00 to 5:00 and 15:00 to 24:00, supplying essential load demand. In contrast, it transitions to charging mode during peak PV generation periods (6:00 to 14:00) with variable charging rates.
- The hydrogen system power (${P}_{H2}$) represents the difference between ${P}_{EL}$ and ${P}_{FC}$. It demonstrates the persistent electrolyzer operation throughout most of the analysis period. That is because of the minimum limit stated in Eq. (8), with consistently positive power flows, indicating hydrogen production. Notably, the system exhibits a singular negative power excursion at 5:00, during which the fuel cell operates in generation mode to convert stored hydrogen into electrical power. During the PV peak period (6:00–14:00), the electrolyzer operates concurrently with battery charging, consuming surplus renewable energy with varying power magnitudes.
- The EVs exhibit bidirectional power exchange capabilities (${P}_{EV}$), operating predominantly in V2G mode during 13:00–23:00 (indicated by negative power values) before transitioning to G2V charging mode during the late evening interval (22:00–24:00); however, their contribution remains modest relative to total load demand.
- The HV operates in V2G mode (${P}_{HV, V2G}$) as a distributed energy resource during 7:00–10:00, although its magnitude remains limited compared to primary renewable generation sources.
- The wind turbine system (${P}_{WT}$) provides relatively steady but marginal generation throughout the day, reflecting its modest installed capacity and limited competitiveness under the prevailing resource conditions in this location. This is consistent with the recorded wind speeds, which ranged between 3.67 and 7.21 m/s (average 5.0 m/s), yielding a wind power output between 1.72 and 15.49 kW (average 5.8 kW).

Finally, the coordinated operation of complementary storage technologies during PV peak periods (6:00–14:00 h) demonstrates the effective utilization of surplus energy and optimized integration of renewable sources while maintaining system reliability during extended low-generation intervals.

### Case study 2 considering LPSP

Another case study is developed considering the variable LPSP. The values of 0.05, 0.1, 0.15, and 0.2 are applied to S5. The optimal sizing outcomes for S5 across different LPSP values are summarized in Table 7.

**Table 7:** Optimal results for S5 considering various LPSP values.

LPSP	$${N}^{PV}$$	$${N}^{WT}$$	$${N}^{BT}$$	$${N}^{FC}$$	$${N}^{EL}$$	$${N}^{HT}$$	ATC ($)
0	993	8	942	29	56	201	308,141.9
0.05	860	5	756	17	52	75	242,830.0
0.1	797	0	652	13	45	137	213,619.2
0.15	738	3	569	9	44	76	187,591.8
0.2	695	2	496	12	43	60	172,221.7

The results in Table 7 clearly demonstrate the strong impact of the LPSP constraint on the optimal sizing and overall economics of the PV–WT–battery–hydrogen MG. The LPSP of zero requires the system to meet load demand at all times, necessitating oversized component capacities and yielding the highest ATC of $308,141.9. Meeting continuous load demand under this configuration requires 942 battery units and 201 HT units, underscoring its substantial storage needs.

As the LPSP constraint is relaxed from 0 to 0.05, a noticeable reduction in system size and cost is observed. Relaxing the reliability constraint to a 5% loss-of-power-supply probability reduces PV capacity and battery storage requirements by approximately 17% and 27%, respectively. Five wind turbine units are incorporated into this optimal configuration. The EL and HT sizes are reduced, indicating a lower reliance on long-term energy storage. Consequently, the ATC declines sharply to $242,830, demonstrating that even a marginal tolerance for supply interruptions yields substantial economic savings.

Further increasing LPSP to 0.1 continues this trend, with PV and battery capacities reduced to 797 and 652 units, respectively, and a noticeable downsizing of the FC, EL, and HT. The absence of WTs indicates that, under moderate reliability requirements, the optimizer prioritizes economically efficient PV and storage configurations over wind generation. The ATC declines further to $213,619.2, confirming that the cost of reliability diminishes progressively as the LPSP increases. At elevated LPSP levels of 0.15 and 0.2, the system shifts toward a more cost-effective design, prioritizing economic efficiency over full load coverage. As a result, PV capacity drops below 750 units, battery storage below 600 units, and H_2_ESS components are reduced proportionally, reflecting the system's relaxed reliability requirements. WTs reappear with small capacity, indicating their role in reducing costs rather than ensuring strict reliability. At LPSP = 0.2, the ATC reaches its minimum of $172,221.5, approximately 44% lower than the fully reliable baseline at LPSP = 0.

**Fig. 17:**
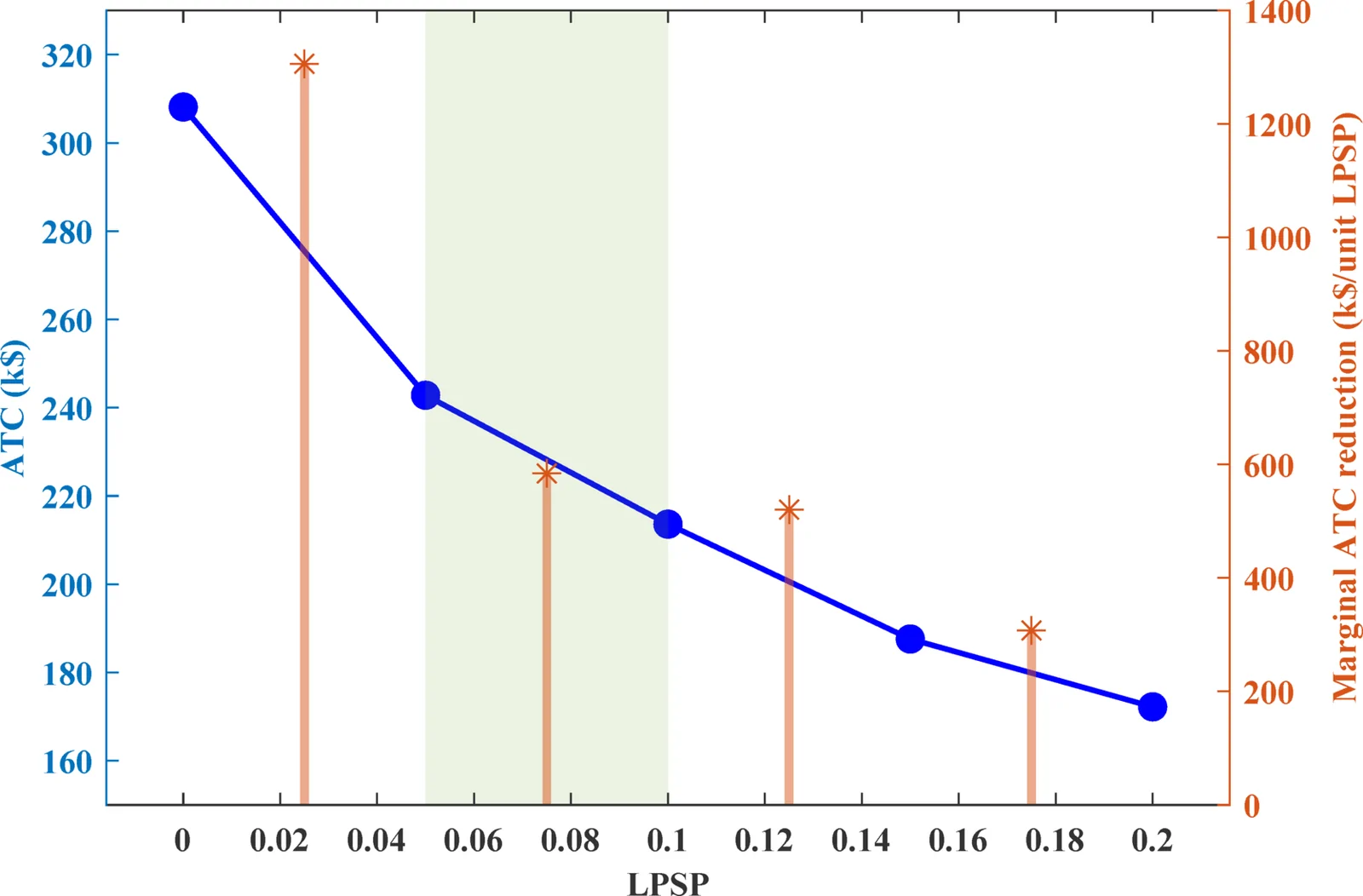
ATC sensitivity to LPSP constraint for scenario S5.

Figure 17 illustrates the ATC-LPSP sensitivity curve, along with the marginal ATC reduction per unit increase in LPSP. The curve exhibits a clear elbow point between LPSP = 0.05 and LPSP = 0.1, where the marginal cost saving diminishes significantly compared to the initial steep decline observed between LPSP = 0 and LPSP = 0.05. This behavior confirms that LPSP = 0.05–0.1, the shaded region, represents the optimal trade-off range, beyond which further relaxation of the reliability constraint yields progressively smaller economic benefits relative to the associated increase in supply interruption risk.

**Fig. 18:**
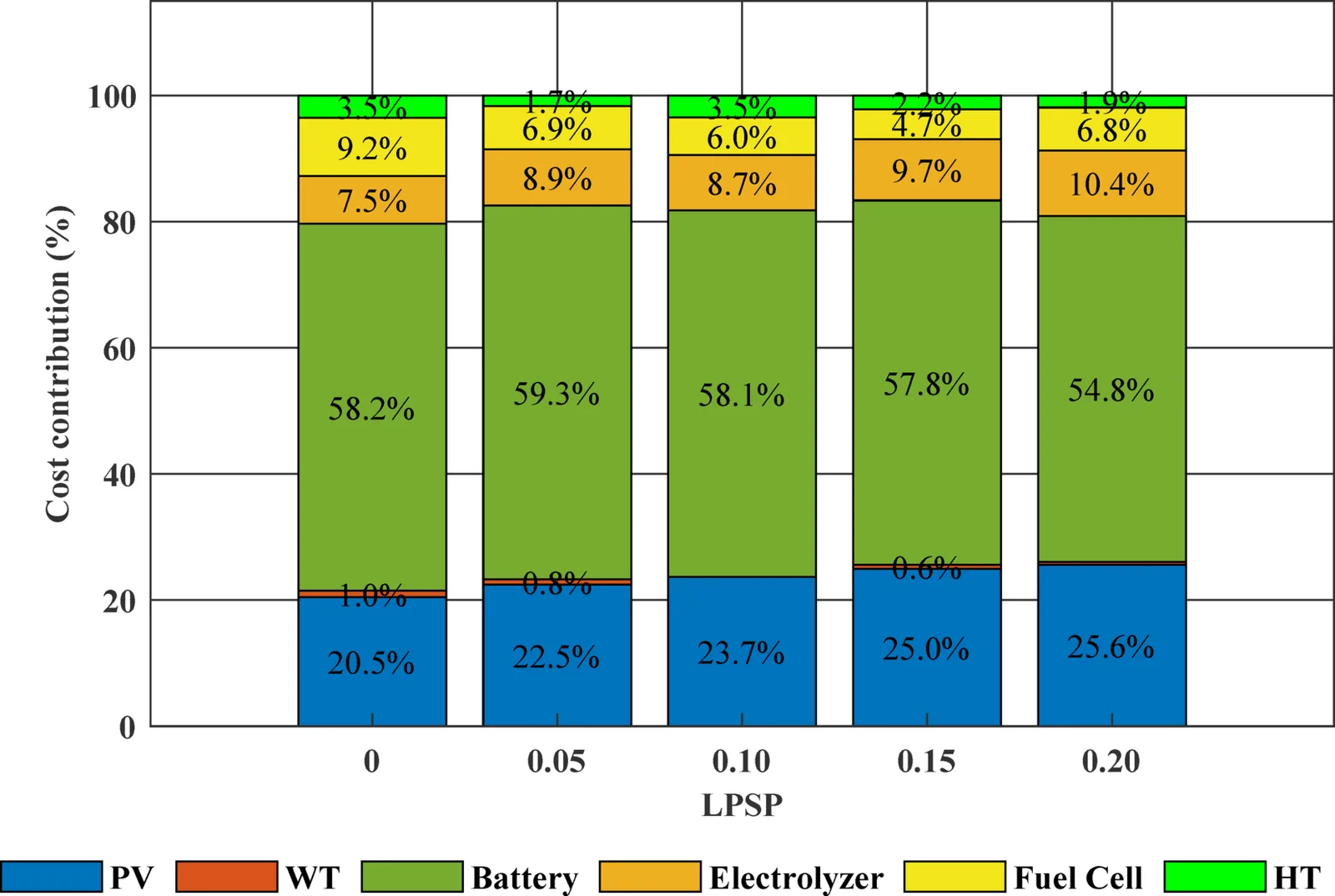
Components’ cost across various LPSP values.

Upon achieving an optimal hybrid energy system by minimizing the ATC across varied LPSP values ranging from 0 to 0.2, Fig. 18 presents a comprehensive cost distribution analysis of MG components. In Fig. 18, the stacked bar chart delineates the percentage contribution of each subsystem, with particular emphasis on hydrogen-based components (EL, FC, and HT). Several critical findings emerge from this economic analysis:

- The battery storage system constitutes the predominant cost component across various LPSP values. It accounts for 54.8–59.3% of total system costs, thereby establishing energy storage as the cornerstone of the hybrid MG architecture. As LPSP values rise from 0 to 0.2, the relative share of battery storage drops from 58.2 to 54.8%. At the same time, the cost of the PV system rises.
- The cost for the PV system demonstrates a marginal upward trend, increasing from 20.5% at LPSP = 0 to 25.6% at LPSP = 0.2. It reflects the trade-off between battery capacity and renewable generation capacity for enhanced system reliability.
- While the EL cost contribution increases from 7.5 to 10.4% as LPSP rises from 0 to 0.2, the combined hydrogen system share declines slightly from 20.2% at LPSP = 0 to 19.1% at LPSP = 0.2. This shift is primarily driven by a decrease in the relative contributions of the FC and HT, which drop from 9.2 to 6.8% and from 3.5 to 1.9%, respectively.
- WT costs remain marginal (0.6–1.0%), corroborating its limited economic viability under the prevailing resource conditions.

These results demonstrate that the primary strategic contribution of H_2_ESS resides in improving reliability and enabling long-duration energy storage rather than reducing overall capital costs. The results indicate that the hydrogen infrastructure is most recognized under the strict reliability standards (LPSP = 0). Therefore, since extended autonomy is required for isolated MGs and cannot be achieved by battery storage alone, the relative investment in hydrogen components is justified for systems that need maximum supply continuity.

## Conclusions

This study addresses the optimal sizing and planning of emission-free rural MGs, accounting for various sources of uncertainty and system variability. The investigated MG consists of renewable sources, a battery, and a green hydrogen system with residential customers as the electric load. Two types of vehicles (EVs and HVs) are modeled in two modes: G2V and V2G. The hydrogen system is used as energy storage and a source for HVs.

Considering the variability in vehicle contributions and operational behavior, the proposed framework with a two-stage optimization approach addresses optimal MG design. The upper layer employs the non-dominated sorting genetic algorithm II to optimize V2G scheduling, thereby minimizing MG load variance and total operational costs for EV and HV owners. The lower-layer approach is a multi-objective optimization problem that minimizes system costs over the entire life cycle while maximizing MG reliability, solved using the Gurobi optimizer.

When both vehicle types are incorporated, multiple scenarios are examined to highlight the effect of V2G mode on optimal sizing. Thus, the following findings are deduced:

- Full G2V and V2G deployment across both HVs and EVs achieves the minimum system cost, reducing total expenditure by 12% relative to the G2V-only scenario.
- The contribution of EVs and HVs in lowering the MG cost is expected to be greater as the number of EVs and HVs increases.

To broaden the analysis, a comprehensive case study is applied to investigate the influence of variable LPSP on optimal sizing under combined G2V and V2G operation of both vehicle types. The study establishes the relationship between supply reliability and optimal MG sizing, providing critical insights for cost-effective system design as follows:

- Strict reliability requires oversized renewable generation and storage capacities, particularly batteries and hydrogen infrastructure, resulting in high system costs.
- On the other hand, allowing higher LPSP values leads to significant reductions in component sizes and ATC. As the LPSP increases, PV generation rises, accompanied by a slight reduction in battery utilization, since the MG requires less energy storage to meet the load demand continuously. Implementing an LPSP constraint of 0.2 reduces the annualized total cost by around 44% compared to the baseline case without the LPSP constraint.
- From a design perspective, the results indicate that moderate LPSP levels (0.05–0.1) offer the optimal compromise between achieving minimum system cost and maintaining acceptable reliability.

### Current limitations

While the proposed framework demonstrated robust performance within the scope of the present study, some limitations should be acknowledged:

- First, the optimal sizing problem was formulated as a mixed-integer quadratically constrained programming problem, involving nonlinear quadratic objective functions, and solved using Gurobi. The computational burden of such problems scales substantially with the temporal resolution and horizon considered. To maintain tractable solution times while still capturing the key seasonal variations affecting renewable generation, demand, and EV/HV mobility patterns, the analysis was conducted over three representative months rather than a full annual horizon. Although this approach is a common and validated practice in MG sizing studies, extending the model to a full year at hourly resolution would substantially increase the problem size and computational time and may be necessary to fully capture rare or extreme seasonal events not represented by the selected months.
- Second, the framework was validated for a single, standalone rural community in El-Kharga Oasis under islanded operation. As such, the results reflect the techno-economic characteristics and resource conditions specific to this location and configuration, and may not directly generalize to grid-connected systems, other geographic regions, or interconnected community clusters without further adaptation.

### Future work

Building on the limitations identified above, several directions are proposed to extend the applicability and generalizability of the proposed framework. The primary direction for future work is applying the framework to multi-community and regional MG clusters. Extending it to networked MG clusters, where neighboring communities can exchange energy, would broaden its applicability to regional electrification planning. Such an extension could also reveal economies of scale in component sizing and hydrogen logistics. However, jointly optimizing multiple interconnected systems would increase computational complexity. Decomposition techniques or distributed optimization approaches could help address this scalability challenge.

Future work could also extend the framework to grid-connected MG configurations, incorporating time-of-use electricity tariffs and net metering. In addition, the stochastic model could be extended to capture renewable energy forecast uncertainty, using scenario-based stochastic programming or robust optimization. Incorporating load demand uncertainty from household behavioral variability would further improve the model, producing more conservative and resilient sizing solutions.

## Data Availability

All data generated or analyzed during this study are included in the article.

## Abbreviations

ATC: Annualized total cost
CI: Capital investment cost
$\mathrm{C}\mathrm{R}\mathrm{F}$: Capital recovery factor
DBO: Dung beetle optimization
DG: Diesel generator
EL: Electrolyzer
ESS: Energy storage systems
EV: Electric vehicle
EVAs: EV aggregators
FC: Fuel cell
FPV: Floating solar photovoltaic
GAMS: General algebraic modeling system
G2V: Grid to vehicle
H_2_: Green hydrogen
H_2_ESS: Hydrogen energy storage system
HHV: High heating value
HRESs: Hybrid renewable energy systems
HT: Hydrogen tank
HV: Hydrogen vehicle
KOA: Kepler optimization algorithm
LPSP: Loss of power supply probability
MFSO: Modified fluid search optimization
MG: Microgrid
MIQCP: Mixed-integer quadratically constrained programming
MT: Micro turbine
NSGA-II: Non-dominated sorting genetic algorithm II
OM: Operation and maintenance cost
OWT: Offshore wind turbines
PDFs: Probability density functions
PSO: Particle swarm optimization
PV: Photovoltaic
RC: Replacement cost
RES: Renewable energy sources
SOC: State of charge
SOH: State of health
STC: Standard test conditions
V2G: Vehicle-to-grid
WT: Wind turbine

## List of symbols

${\mathrm{A}}_{\mathrm{i},\mathrm{t}}^{\mathrm{E}\mathrm{V}}$: Status of *i* th EV (charging or discharging) at time $\mathrm{t}$
${A}_{i,t}^{HV}$: Status of *i* th HV in G2V mode
${C}_{j}^{CI}$: Capital investment cost of component *j*
${C}_{j}^{OM}$: Operation and maintenance cost of component *j*
${C}_{j}^{RC}$: Replacement cost of component *j*
${C}_{V2G}^{EV}$: EV loss cost during V2G mode
${C}_{V2G}^{HV}$: HV loss cost during V2G mode
$${\mathrm{c}}^{\mathrm{B}\mathrm{a}\mathrm{t}}$$: Battery cost
$${c}^{FC}$$: FC cost
${\mathrm{c}}_{\mathrm{t}}^{\mathrm{E}\mathrm{V}}$: Charging and discharging tariffs for EVs at time *t*
${c}_{t}^{HV}$: Charging and discharging tariffs for HVs at time *t*
*D*: WT rotor diameter
${\mathrm{d}}_{\mathrm{m}\mathrm{a}\mathrm{x}}^{\mathrm{H}\mathrm{V}}$: Maximum distance for HV
${\mathrm{E}}_{\mathrm{t}}^{\mathrm{H}\mathrm{V}}$: Energy consumed by the HV at time *t*
${\Delta \mathrm{E}}^{\mathrm{E}\mathrm{V}}$: Energy consumption per km for EV
${\Delta \mathrm{E}}^{\mathrm{H}\mathrm{V}}$: Energy consumption per km for HV
${G}_{\mathrm{t}}$: Actual solar irradiance intensity at time *t*
${G}_{STC}$: Solar irradiance at STC
${H}_{ref}$: Wind reference height
$${H}_{WT}$$: WT height
${m}_{r}^{HT}$: Rated capacity of HT
${\mathrm{m}}_{\mathrm{t}}^{\mathrm{c}\mathrm{h}}$: Charging mass for each HV at time *t*
${m}_{t}^{dis}$: Discharging mass for each HV at time *t*
*N*: Project lifespan
${N}^{j}$: Number of units used of component *j*
*n*: Maximum number of battery cycle count for an EV
${V}_{ci}$: Cut-in wind speed
${V}_{co}$: Cut-out wind speed
${V}_{r}$: Rated wind speed
*α *: Friction coefficient
${\delta}_{BT}^{Dsp}$: Lost power during discharging and charging
${\eta }^{j}$: Efficiency of component *j*
${\eta }^{PV}$: Power loss coefficient for PV panels
${\upmu}_{\mathrm{d}}^{\mathrm{E}\mathrm{V}}$,${\upmu}_{\mathrm{t}\mathrm{a}}^{\mathrm{E}\mathrm{V}}$, ${\upmu}_{\mathrm{t}\mathrm{d}}^{\mathrm{E}\mathrm{V}}$: Mean value for driving distance, arrival, and departure time for EV
${\upmu}_{{\mathrm{T}}_{\mathrm{a}}}$, ${\updelta}_{{\mathrm{T}}_{\mathrm{a}}}$: Mean value and standard deviation for the arrival time of HV
${\upmu}_{{\mathrm{m}}_{\mathrm{i}\mathrm{n}}}$*,*${\updelta}_{{\mathrm{m}}_{\mathrm{i}\mathrm{n}}}$: Mean value and standard deviation for the initial state of charging of HV
*φ *: Power temperature coefficient
${\updelta}_{\mathrm{d}}$, ${\updelta}_{{\mathrm{t}}_{\mathrm{a}}}$, ${\updelta}_{{\mathrm{t}}_{\mathrm{d}}}$: Standard deviation for driving distance, arrival, and departure time for EV
